# Anionic lipids modulate the membrane localization and conformational dynamics of KirBac1.1 slide helix during lipid-dependent activation

**DOI:** 10.1042/BCJ20253215

**Published:** 2025-09-30

**Authors:** Arpan Bysack, Chandrima Jash, H. Raghuraman

**Affiliations:** 1 Crystallography and Molecular Biology Division, Saha Institute of Nuclear Physics, Kolkata, India; 2 Homi Bhabha National Institute, Training School Complex, Mumbai, India

**Keywords:** hydration dynamics, inward-rectifier K^+^channel, lipid-dependent gating, liposome flux assay, site-directed fluorescence

## Abstract

Inward-rectifier potassium (Kir) channels are essential for regulating various physiological processes and are implicated in several life-threatening diseases, making them key drug targets. KirBac1.1, a well-characterized prokaryotic homolog of Kir channels, is known to undergo anionic lipid-dependent gating. Although the slide helix is an important structural component in the gating mechanism of KirBac1.1, its structural dynamics associated with the anionic lipid-driven activation is not well understood. Here, we have reconstituted KirBac1.1 in zwitterionic 1-palmitoyl-2-oleoyl-*sn*-glycero-3-phosphocholine (POPC) and anionic POPC/ 1-palmitoyl-2-oleoyl-sn-glycero-3-phospho-(1′-rac-glycerol) (sodium salt) (POPG) membranes to stabilize the inactive and active conformations of the channel, respectively. Our liposome K^+^ flux assay results show that all the slide helix single-cysteine mutants display PG-driven gating, and increasing the PG from 25 to 40 mol% does not have any linear dependency on both the activation and K^+^ flux rates. Site-directed 7-nitrobenz-2-oxa-1,3,-diazol-4-yl (NBD) fluorescence results suggest that the structural dynamics of the slide helix is significantly altered upon PG-induced activation. For instance, we observe significant changes in hydration dynamics and rotational mobility of slide helix residues between functional states. Maximum entropy method-based lifetime distribution analysis suggests that the conformational heterogeneity of the slide helix is functional-state dependent. Importantly, membrane penetration depth measurements reveal that the slide helix in the active KirBac1.1 is located ~3 Å deeper within the membrane interface, well supported by increased fluorescence lifetimes. Notably, the non-linear relationship between structural dynamics and PG content highlights the critical role of lipid-protein interactions and membrane surface charge in PG-mediated KirBac1.1 activation. These findings provide valuable insights into Kir channel gating mechanisms and lipid-dependent gating of other channels.

## Introduction

Potassium (K^+^) channels are widely expressed and play an essential role in action potential generation amongst multiple physiological processes [1,2]. Mutations in human K^+^ channels are linked to a plethora of diseases, which include epilepsy, deafness, arrhythmias, periodic paralysis, hypertension, hyperinsulinaemic hypoglycaemia and Andersen syndrome [3]. In particular, the homotetrameric eukaryotic inward-rectifier K^+^ (Kir) channels are an important class of K^+^ channels that regulate membrane excitability, heart rate, vascular tone, insulin secretion and epithelial salt transport, and are implicated in various forms of neuronal, cardiac and kidney diseases [4,5]. They exhibit inward rectification, meaning they allow more K^+^ influx than efflux due to voltage-dependent pore blockage by cytoplasmic polyamines and Mg²^+^. There are seven Kir subfamilies (Kir1-7) containing 16 human Kir channels, which differ in rectification strength and regulatory mechanisms [5–7].

KirBac1.1 is the widely studied prokaryotic homolog of Kir channels. It shares ~50% sequence homology with mammalian Kir channels and has been shown to be a pukka inward-rectifying K^+^ channel [8]. This bacterial homolog is, therefore, instrumental in elucidating molecular mechanisms governing Kir channel gating-related conformational changes [8–16]. The crystal structure of KirBac1.1 from *B. pseudomallei* provides a high-resolution structural model of the closed state, which is common to all Kir channels [17]. Despite solving the closed state conformation of KirBac1.1 more than 20 years ago in detergent micelles, the high-resolution structural models of the open and other functional states are still elusive. KirBac1.1 is a homotetramer and consists of transmembrane α-helical pore domain (each subunit has 2 α-helices) and a cytosolic domain consisting predominantly of β-sheets. With respect to the pore domain, it shares most of the structural features with KcsA K^+^ channel [6,18–21], i.e., it has an outer vestibule, selectivity filter, pore helix and a N-terminal amphipathic helix, which is termed ‘slide helix’ in KirBac1.1. It has recently been shown that organization and dynamics of the slide helix of the closed conformation is significantly altered in micelles and membranes [22].

KirBac1.1 function is highly dependent on lipid composition in the sense that it undergoes lipid-dependent gating. For instance, while the closed/inactive conformation of KirBac1.1 is stabilized in zwitterionic membranes [15,22], the presence of negatively charged lipids stabilizes the open/active conformation [9,14–16]. The slide helix of KirBac1.1 interacts with the anionic lipids at the cytoplasmic interface to regulate channel activity. Notably, the slide helix residues play an important role in the mechanism of cooperative gating in which KirBac1.1 undergoes structural transition from closed/inactive to open/active state [9,14,16]. The slide helix is, therefore, a crucial structural motif for modulating the channel activity. However, the structural dynamics of the slide helix associated with the gating-related structural transition in membranes is not fully understood.

In this study, we have probed the structural dynamics of the functionally pertinent slide helix in different functional states utilizing the single-cysteine mutants of the slide helix and various site-directed fluorescence approaches [23,24]. Our liposome K^+^ flux assay results suggest that the K^+^ transport activity of slide helix mutants is not linearly dependent on increasing membrane surface charge. Results from site-directed 7-nitrobenz-2-oxa-1,3,-diazol-4-yl (NBD) fluorescence approaches suggest that the structural dynamics of the slide helix is significantly altered between the inactive and active conformations of the channel. For instance, membrane penetration depth analysis indicates that the slide helix of the active conformation of KirBac1.1 resides deeper at the membrane interface, supported by increased fluorescence lifetimes. Our results also show that the hydration dynamics and rotational mobility of the slide helix residues are significantly altered in different functional states. The conformational heterogeneity of the slide helix, as monitored by the lifetime distribution analysis, appears to be functional-state dependent. Taken together, these results demonstrate anionic lipid 1-palmitoyl-2-oleoyl-sn-glycero-3-phospho-(1′-rac-glycerol) (sodium salt) (POPG)-dependent structural reorganization of the slide helix at the membrane interface during KirBac1.1 activation. Collectively, our findings underscore the importance of lipid–protein interactions in membranes and could advance our understanding of not only Kir channel gating but also other channels that undergo lipid-dependent gating.

## Results

### Secondary structure of the NBD-labelled KirBac1.1 analogues in zwitterionic and anionic membranes

**Figure 1:**
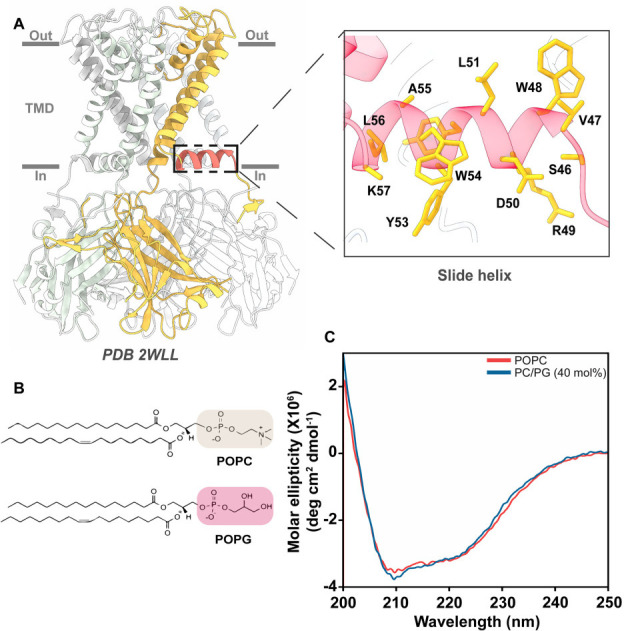
The slide helix in the closed state of KirBac1.1. (**A**) Shown is the cartoon representation of closed state crystal structure of homotetrameric KirBac1.1 (*PDB: 2WLL*) highlighting one of the subunits of the tetramer in which the slide helix (shown in red) is indicated by a dotted box. The inset shows the enlarged view of the slide helix and its native amino acid residues in stick representation. The membrane boundaries are arbitrarily depicted as lines. (**B**) Chemical structures of zwitterionic phospholipid POPC and anionic phospholipid POPG are shown. The headgroups of the lipids are highlighted. (**C**) Representative far-UV CD spectra of 5 μM NBD-labelled W54C mutant of KirBac1.1 reconstituted in POPC and PC/PG (3:2 mol/mol) membranes are shown.

To probe the structural dynamics of the slide helix (residues ^46^SVWRDLYYWALK^57^) of KirBac1.1 (Figure 1A) in different functional states utilizing site-directed fluorescence approaches, 12 single-cysteine mutants were generated. The single-cysteine mutants of KirBac1.1 are independently purified and labelled with a thiol-reactive, environment-sensitive fluorophore NBD, which has been widely used to monitor the dynamics of membrane-mimetic systems [25–27] and structural dynamics of several classes of membrane proteins [20,22–24,28–32]. We have recently shown that the NBD-labelled slide helix analogues are structurally similar to wildtype channel in DM micelles [22]. Upon reconstitution of the NBD-labelled slide helix mutants of KirBac1.1 in membranes composed of zwitterionic 1-palmitoyl-2-oleoyl-*sn*-glycero-3-phosphocholine (POPC) and anionic POPC/POPG (3:2, mol/mol) lipids (Figure 1B), the predominantly α-helical nature of KirBac1.1 remains similar in all cases (W54-NBD is shown as representative) as seen from the far-UV circular dichroism (CD) spectra (Figure 1C). This suggests that the secondary structure of the KirBac1.1 is not affected by the increased membrane surface charge.

### The single-cysteine mutants of the slide helix are active in POPC/POPG membranes

**Figure 2:**
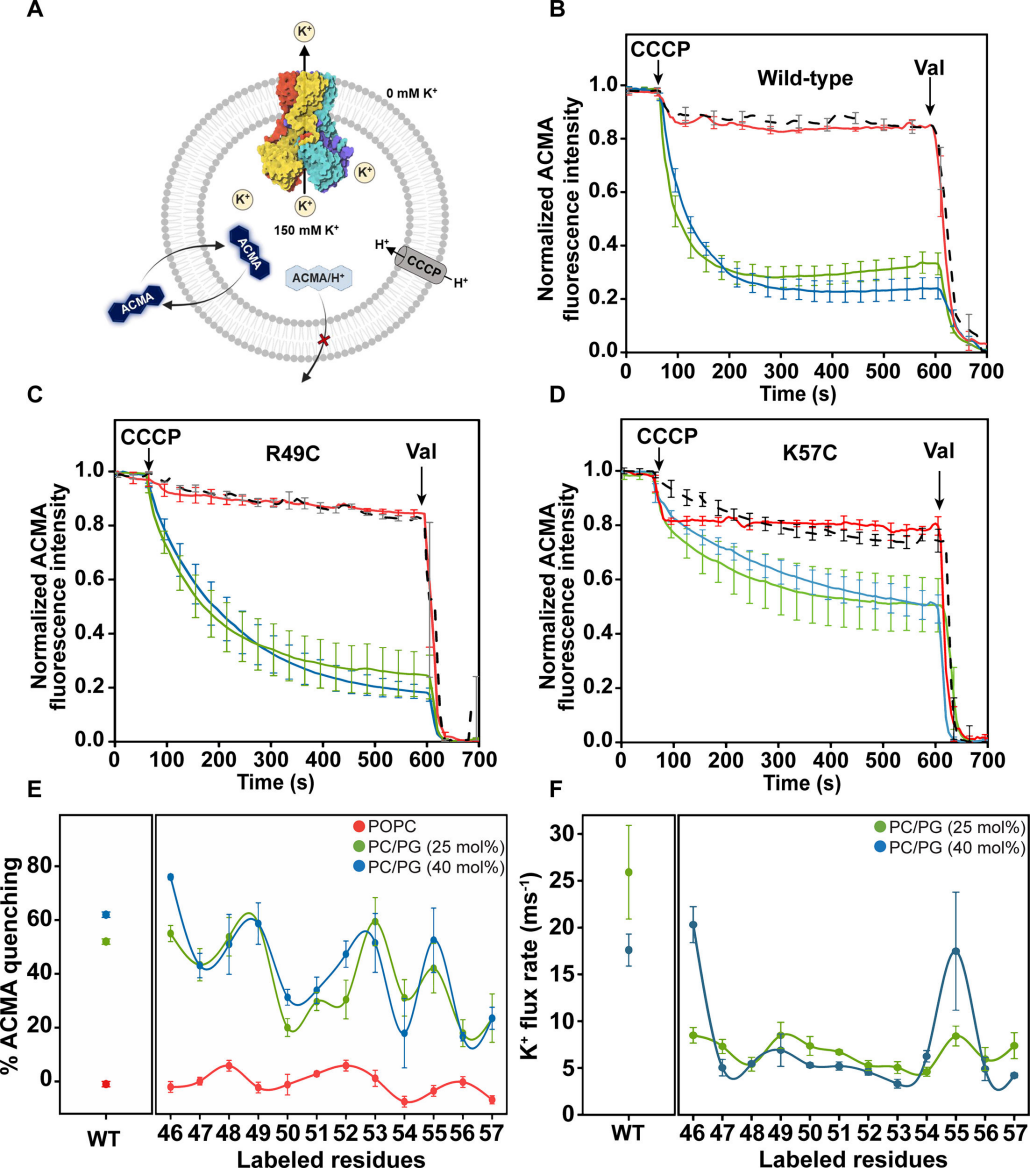
Single-cysteine mutants of the slide helix are active in PG-containing membranes. (**A**) Schematic representation of K^+^ transport assay in liposomes using ACMA fluorescence is shown. K^+^ transport assay of (**B**) wildtype KirBac1.1, (**C**) R49C, and (**D**) K57C mutants in POPC (red trace), and POPC/POPG membranes containing 25 mol% (*i.e*. 3:1 molar ratio, green trace) and 40 mol% (i.e. 3:2 molar ratio, blue trace) POPG lipid. Shown are the changes in the normalized fluorescence intensity of pH-sensitive dye ACMA monitored in real time. Empty liposomes (i.e. without protein) serve as control (black trace). The excitation wavelength used was 410 nm and the emission was monitored at 480 nm. Arrows show the time at which CCCP and valinomycin were added. All data are represented as mean ± SE of three independent measurements. Data shown for wildtype channel in POPC membranes are taken from [22] for comparison. (**E**) The percentage ACMA quenching for wild-type and slide helix mutants reconstituted in POPC and POPC/POPG liposomes with varied PG content are shown. (**F**) The K^+^ flux rates, obtained by monoexponential decay fit of ACMA quenching traces for the slide helix mutants in PC/PG membranes are shown. The lines joining the data points in (**E**) and (**F**) are provided merely as viewing guides. See Materials and Methods and text for other details.

Using the stability mutant of KirBac1.1 (I131C), Wylie’s group has demonstrated that anionic lipids are essential for the function of KirBac1.1 and the channel remains in the inactive/closed conformation in zwitterionic POPC membranes [15,16]. We have recently shown that wildtype KirBac1.1 and the single-cysteine mutants of the slide helix do not transport K^+^ in zwitterionic POPC membranes due to their stabilization in the inactive/closed conformation [22]. To ensure that the wildtype and single-cysteine slide helix mutants of KirBac1.1 are in the active/open conformation in POPC/POPG membranes, we have performed K^+^ transport activity using the 9-amino-6-chloro-2-methoxyacridine (ACMA)-based liposome flux assay [33,34]. In this assay (Figure 2A), when the protein-reconstituted liposomes loaded with K^+^ are incubated with the membrane-permeable pH-sensitive ACMA fluorophore, the addition of the proton ionophore CCCP results in quenching of ACMA fluorescence if the channel is in active/open conformation, i.e*.,* H^+^ enters via CCCP if the channel performs outward conduction of K^+^. Since the protonated ACMA loses its fluorescence and becomes membrane-impermeable, this results in the decrease of ACMA fluorescence, i.e., quenching. The extent of ACMA quenching is directly related to the relative K^+^ transport activity. This assay has been widely used to monitor the transport activity of potassium, sodium, proton and chloride channels [14–16,33–39].

Our results show that wildtype KirBac1.1 exhibits K^+^ transport function in POPC/POPG (3:1 and 3:2, mol/mol) membranes containing 25 and 40 mol% phosphatidylglycerol (PG) lipid, respectively (Figure 2B), which is in excellent agreement with the previous results [15,16]. Representative ACMA fluorescence quenching traces for R49C (Figure 2C) and K57C (Figure 2D) mutants in POPC and POPC/POPG membranes are shown. Importantly, all single-cysteine slide helix mutants show significant ACMA quenching, i.e., varied degrees of K^+^ transport activity in POPG-containing membranes (Figure 2E). This suggests that the single-cysteine slide helix mutants of the channel are in active/open conformation in anionic membranes.

Interestingly, comparison of all the functional data of wildtype and slide helix mutants of KirBac1.1 shows that although anionic lipid POPG activates the channel, increasing the PG content from 25 to 40 mol% does not have any linear dependency on the activation (Figure 2E) as well as the K^+^ flux rate (Figure 2F). For instance, in a wildtype channel, increasing the PG content in the membrane decreases the relative K^+^ flux rate from ~27 ms^−1^ to 16 ms^−1^. It should be noted that varied flux rates of K^+^ are reported for the I131C stability mutant of KirBac1.1 in different membrane composition containing 25 mol% PG. In PE/PG and PC/PG membranes, the reported K^+^ flux rates are ~30 ms^−1^ [14] and ~14 ms^−1^ [16], respectively. Nevertheless, our K^+^ flux rates for the wildtype channel are in overall agreement with the stability mutant of KirBac1.1. In the case of slide helix single-cysteine mutants, the K^+^ flux rates are in the range of ~5–20 ms^−1^ in both 25 mol% and 40 mol% PG containing membranes, and there is no obvious pattern upon increasing the PG content from 25 to 40 mol%. Considering the reported EC_50_ (flux rate vs. increasing PG mol%) value of ~21% PG [16], it is possible that we are already in the saturating effects of PG-mediated gating of KirBac1.1. Although the %ACMA quenching for many of the slide helix mutants is similar to wildtype (Figure 2E), the rates are considerably reduced (Figure 2F). This is because %ACMA quenching denotes the overall K^+^ transport activity, whereas the K^+^ transport rates are dependent on the slope of the quenching traces and are calculated from the mono-exponential decay fit of the same. Nevertheless, our functional results demonstrate that, like wildtype KirBac1.1, the single-cysteine slide helix mutants also display anionic lipid-driven gating of the channel. Importantly, the NBD-labelled single-cysteine mutants of the channel are also in active/open conformation in anionic membranes (see Supplementary Figure S1). This is helpful in comparing the structural dynamics of the functionally important slide helix in closed (in POPC membranes) and open conformation (in POPC/POPG membranes) of the KirBac1.1.

### Local environment of the slide helix in the active/open conformation of KirBac1.1

**Figure 3:**
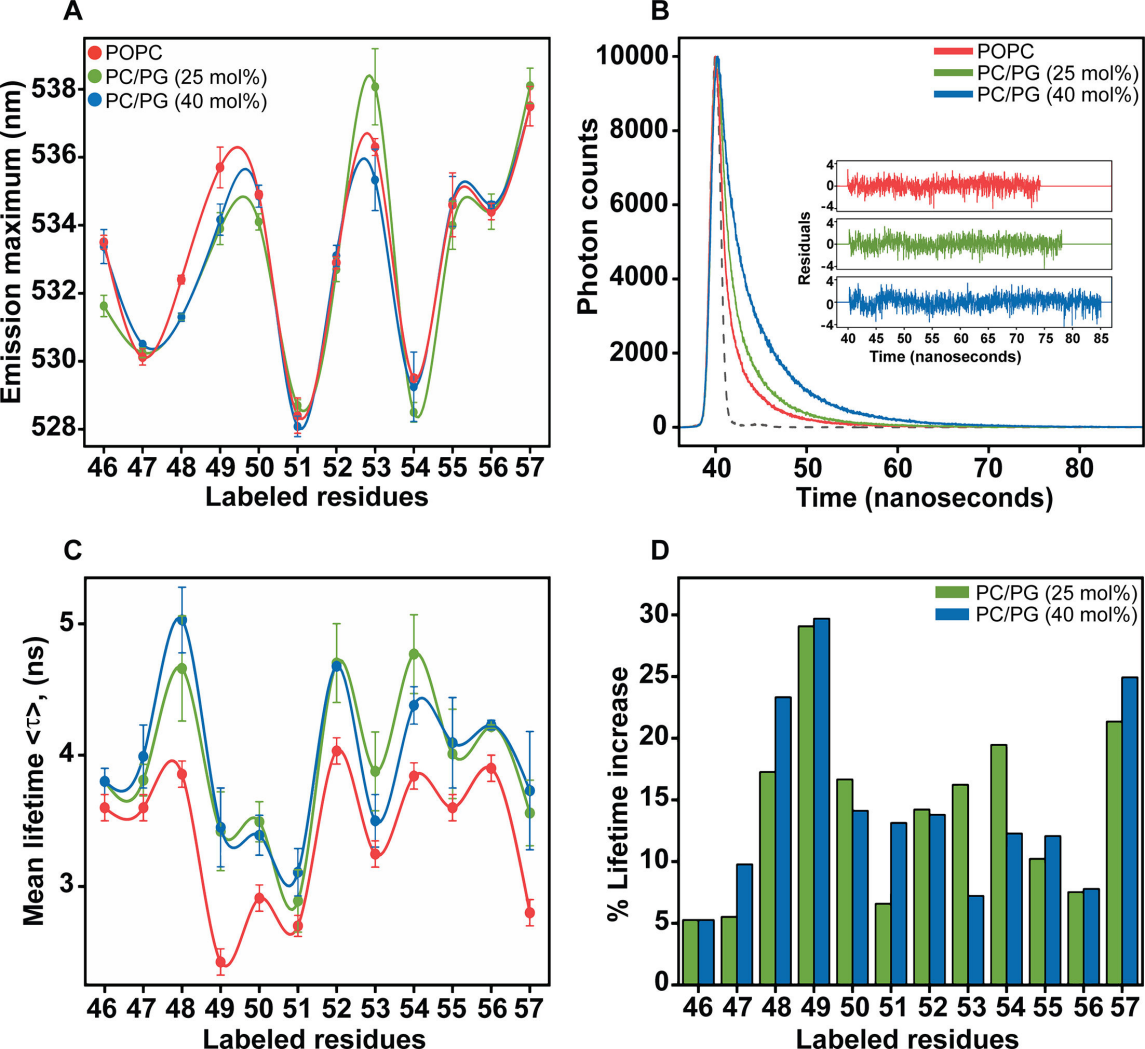
Microenvironment of slide helix residues in the active/open state of KirBac1.1. (**A**) Fluorescence emission maximum, (**B**) time-resolved fluorescence intensity decay profile (W48-NBD shown as representative), and (**C**) mean fluorescence lifetimes of the NBD-labelled slide helix residues in POPC (red), and POPC/POPG membranes containing 25 mol% (green) and 40 mol% (blue) POPG lipid are shown. In (**B**), the dotted grey trace is the lamp profile and the fluorescence decay profiles of W48-NBD in various membranes, fitted with a triexponential function, are shown along with the corresponding weighted residues of the respective decay fit in the inset. Excitation wavelength was 456 nm, and emission was monitored at respective emission maximum. In (**A**) and (**C**), data represent mean ± SE of three independent measurements, and the lines joining the data points are provided merely as viewing guides. The concentration of KirBac1.1 was 1.6 μM and the protein/lipid molar ratio of 1:100 in all cases. Data shown for slide helix residues in POPC membranes are taken from [22] for comparison. (**D**) The effect of increasing anionic POPG lipid (25 mol, green) and 40 mol%, blue) in membranes on the lifetimes of labelled mutants is shown as % lifetime increase with respect to zwitterionic POPC.

It is well documented that the fluorescence of NBD shows drastic changes upon change in the polarity of the medium, i.e., it is highly environment-sensitive [23,40,41]. This property has been effectively used to probe the location of NBD-labelled residues in different environments, namely the aqueous phase, interfacial region or the hydrophobic core in membranes [23,28]. We have recently characterized the organization and dynamics of the slide helix in POPC membranes and showed that the slide helix in the inactive/closed state of the channel is not fully exposed to aqueous milieu; rather, it resides at the interfacial region of POPC membranes [22]. Here, we monitor the effect of the presence of anionic lipid POPG in membranes on the organization and structural dynamics of the slide helix in the active conformation of the channel. The fluorescence emission maxima of the NBD-labelled slide helix residues are similar in both functional states of KirBac1.1, ranging from ~528 to 536 nm and ~528 to 538 nm in POPC and PC/PG membranes, respectively, depending on the content of PG (Figure 3A). The magnitude of emission maximum values suggests that the slide helix is partitioned in membranes [27], and most of the residues of the slide helix are located at chemically heterogeneous interfacial region in both cases.

Since fluorescence lifetime is an intrinsic property of the fluorophore and serves as a reliable indicator of the local environment [23,24,42], we measured the fluorescence lifetimes of the NBD-labelled slide helix residues using the TCSPC mode to monitor the microenvironment of the NBD-labelled residues of the slide helix. Typical decay profiles of W48-NBD in POPC, PC/PG (3:1, mol/mol), and PC/PG (3:2, mol/mol) membranes with its tri-exponential fitting and the residuals to check the goodness of fit are shown in Figure 3B. The pre-exponentials and the corresponding lifetimes of the triexponential fit for all the slide helix residues are shown in Supplementary Table S1.

The intensity-weighted mean fluorescence lifetimes, <τ>, of NBD-labelled slide helix in inactive (POPC) and active (3:1 and 3:2 POPC/POPG) conformations are shown in Figure 3C. The trend observed for the variation in fluorescence lifetime is similar in all cases, suggesting the overall structure of the slide helix is not significantly changed in both functional states of KirBac1.1. While the fluorescence lifetimes of the NBD-labelled slide helix residues in POPC membranes are in the range of ~2.5–4 ns, the corresponding range is ~3–5 ns in PC/PG membranes, suggesting that the slide helix residues exhibit increased lifetimes in POPG-containing membranes (Figure 3D). Since decreased microenvironment polarity around the NBD group results in blue-shifted emission maximum and significantly increased lifetime [26], this profound increase in lifetime (~5–30%) could be due to partitioning of the slide helix at the deeper interfacial region of the membrane in the active state of the channel, which would result in decreased water penetration.

It should be noted that the general notion of decreased environmental polarity would result in increased lifetimes and blue-shifted emission maximum is not applicable to a few slide helix residues, probably due to complex interaction network with the neighbouring residues. For instance, the lifetime of L51-NBD is unexpectedly low in both POPC and PC/PG membranes despite exhibiting the most blue-shifted emission maximum of ~528 nm (see Figure 3, A and C). Similarly, Y53-NBD shows a red-shifted emission maximum in the presence of anionic lipid, yet its lifetime is considerably increased. This anomaly could be attributed to either increased aqueous exposure or due to the presence of nearby residue(s) that can act as a quencher of NBD fluorescence and facilitate the decay process. We believe the latter might be the predominant factor since the water penetration around the slide helix is not only significantly decreased but also the presence of charged and/or aromatic residues (Trp or Tyr) within 4 Å of the slide helix residues (see Supplementary Figure S2), which can quench the fluorescence of extrinsic fluorophores [43,44]. Overall, these results probably suggest PG-driven conformational changes of the slide helix within the membrane when the channel shuttles from inactive to active conformation.

### Rotational dynamics of the slide helix in the active/open conformation of KirBac1.1

**Figure 4:**
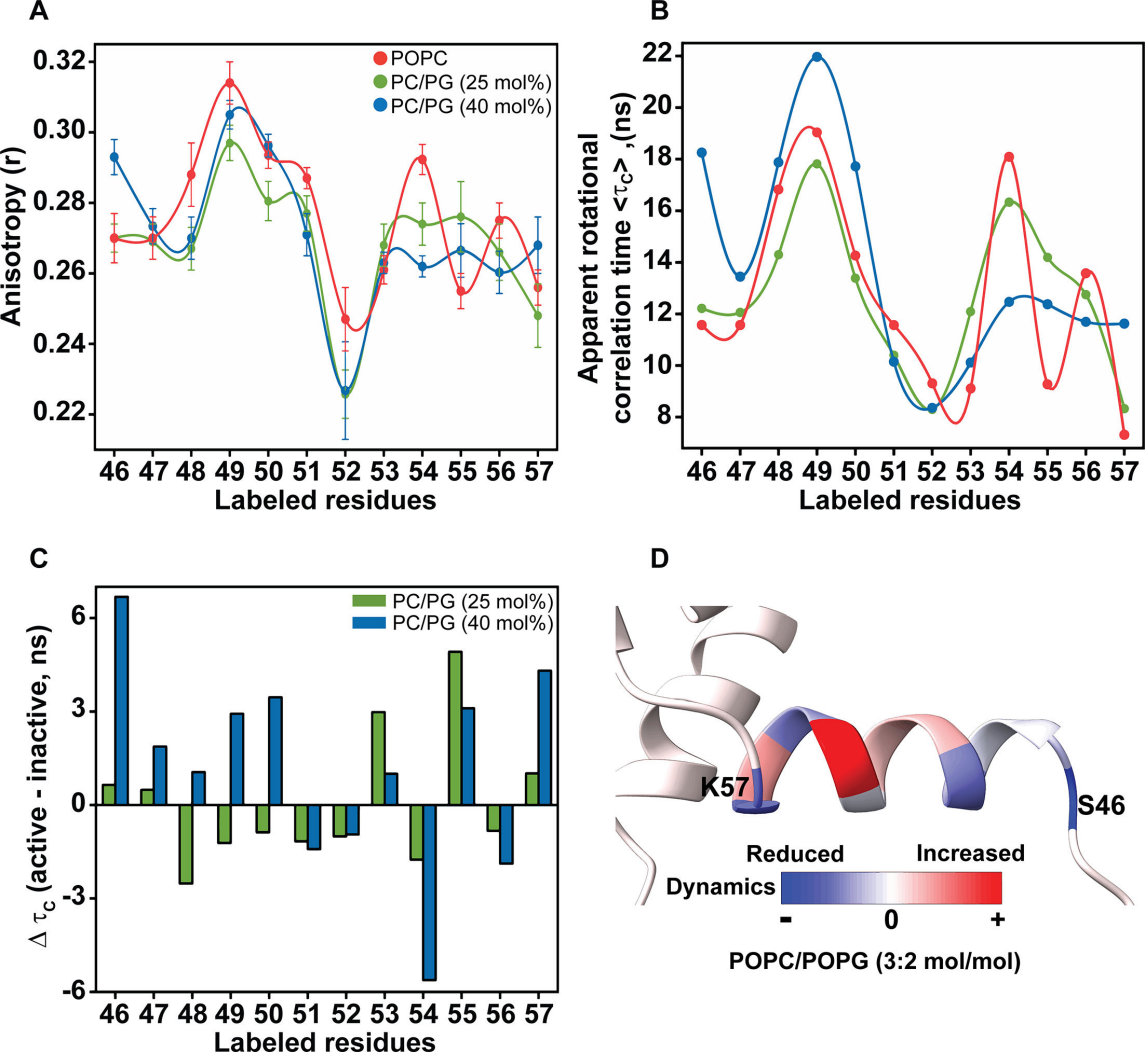
Rotational dynamics of slide helix residues in membranes. (**A**) Steady-state anisotropy of NBD-labelled slide helix residues reconstituted in POPC liposomes (red) and PC/PG liposomes with 25 mol% (green) and 40 mol% PG lipid (blue) at a protein/lipid molar ratio of 1:100. The excitation wavelength used was 465 nm and the concentration of KirBac1.1 was 1.6 μM in all cases. Data represent mean ± SE of three independent measurements, and the lines joining the data points are provided merely as viewing guides. (**B**) Shown are the calculated apparent rotational correlation times (τ_c_) of the slide helix mutants in POPC liposomes (red) and PC/PG liposomes with 25 mol% (green) and 40 mol% PG lipid (blue). (**C**) The differences in τ_c_ (Δτ_c_) between active (in PC/PG) and inactive (in POPC) conformations are shown. (**D**) Δτ_c_ values between 40 mol% PG containing membranes and POPC were mapped on the crystal structure of KirBac1.1 (*PDB: 2WLL*) to highlight the PG-dependent gating induced changes in dynamics of the slide helix. Data for the inactive state (i.e. in POPC membranes) were adapted from [22]. See Materials and Methods for other detail

Fluorescence anisotropy is one of the powerful site-directed fluorescence approaches to monitor the molecular flexibility and rotational motion of a fluorophore [45] and has been used to probe gating-related dynamic behaviour of several classes of ion channels [20,23,24,46,47]. It is well known that the rapidly tumbling NBD displays negligible anisotropy, whereas its limiting anisotropy is 0.354 [24,25]. The steady-state fluorescence anisotropy values of NBD-labelled slide helix residues in POPC and PC/PG membranes, which are in the range of ~0.23–0.31, suggest that the slide helix displays high dynamic variability (Figure 4A). This, in general, indicates that the rotational mobility of the NBD-labelled slide helix is considerably restricted.

As shown above, slide helix residues in PC/PG membranes have increased lifetimes compared with POPC membranes. To ensure that the measured anisotropy values for the NBD-labelled slide helix residues of KirBac1.1 do not suffer from lifetime-induced artefacts, apparent rotational correlation times were calculated and are shown in Figure 4B. As can be seen from the figure, the rotational correlation times (τ_c_), on average, for the slide helix residues of the channel in both POPC and PC/PG membranes containing 40 mol% PG are ~8–22 ns, supporting that the slide helix exhibits high dynamic variability in these membranes. Differences in τ_c_ (i.e. Δτ_c_) values between active (in PC/PG) and inactive (in POPC) conformations (Figure 4C) reveal that the PG-induced slide helix rotational dynamics is very complex in nature. Compared with its inactive conformation in POPC membranes, it appears that the N-terminal half of the slide helix is slightly dynamic in the presence of 25 mol% PG. However, barring residue 48, the rotational dynamics changes at this region of the slide helix are modest. Further increase of PG content to 40 mol% in membranes makes most of the slide helix residues undergo restricted mobility (i.e. less dynamic). The restricted mobility of the slide helix at high concentration of anionic lipid PG in membranes could be attributed to the presence of charged residues (particularly R49), which might increase hydrogen bonding ability and electrostatic interactions leading to its overall restricted rotational dynamics while stabilizing the active conformation of the channel. Interestingly, irrespective of the PG content, the C-terminal half of the slide helix undergoes dynamic variability. Mapping the Δτ_c_ values between the active (at 40 mol% PG) and inactive conformations (Figure 4D) in the structure of slide helix suggests differential dynamics throughout the slide helix in PG-containing membranes. The N-terminal half of the slide helix (residues 46–50) experiences restricted mobility, whereas the C-terminal half (residues 51–57) does not have a definite pattern in the sense that the mobility fluctuates a lot in this region upon PG binding. Taken together, the significantly altered rotational mobility in the presence of anionic lipids suggests PG-dependent structural reorganization of the slide helix when KirBac1.1 transitions into the active conformation.

### Changes in water accessibility of slide helix in different functional states of KirBac1.1

**Figure 5:**
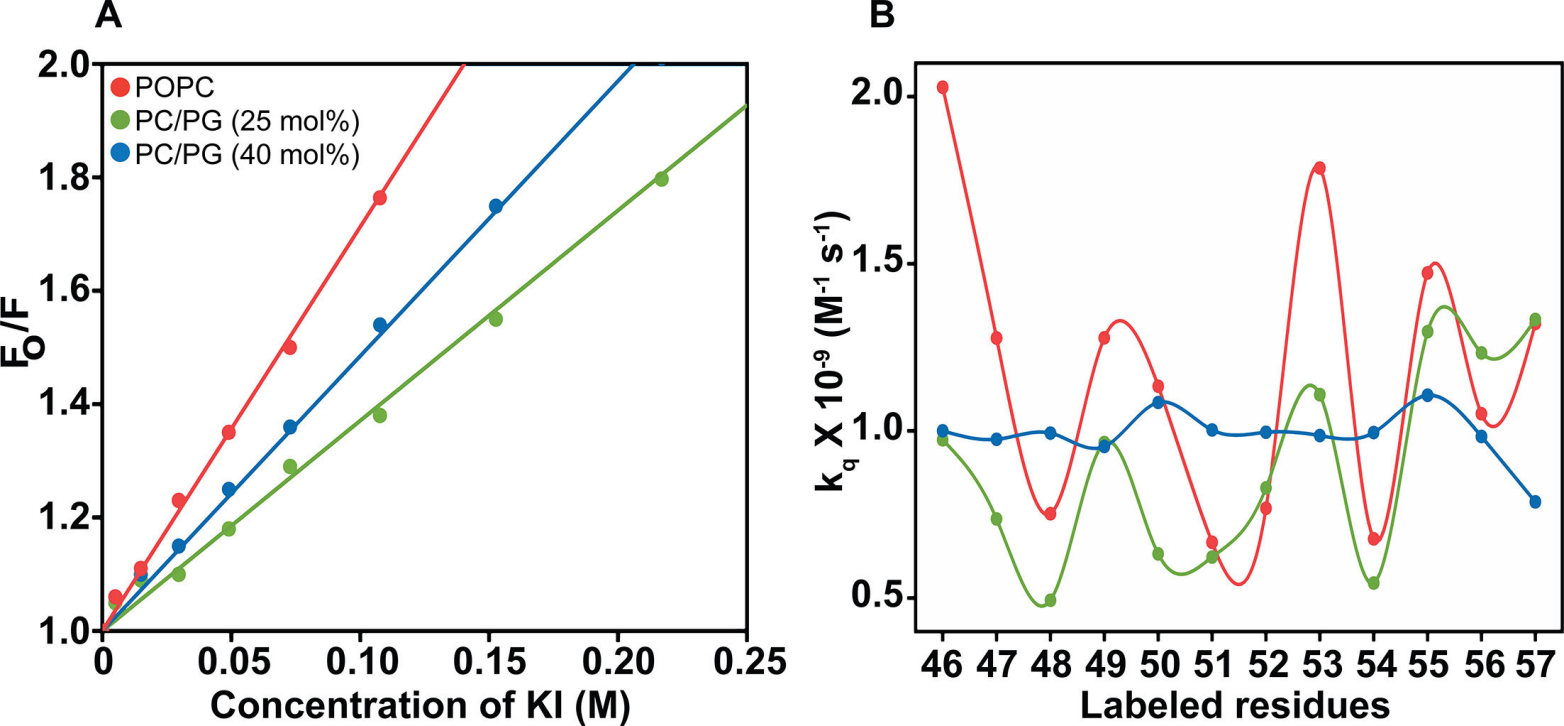
Water accessibility probed by iodide quenching of NBD ﬂuorescence. (**A**) Shown are representative data for Stern-Volmer analysis of iodide quenching in POPC liposomes (red) and PC/PG liposomes at 3:1 (green) and 3:2 (blue) molar ratios for S46-NBD of the slide helix. *F*_o_ is the fluorescence intensity in the absence of quencher, *F* is the corrected fluorescence in the presence of quencher. The excitation wavelength used was 465 nm, and the emission was monitored at respective emission maximum. The concentration of protein was 1.6 μM and the protein/lipid molar ratio is 1:100 in all cases. (**B**) The calculated bimolecular quenching constants (k_q_), using the mean K_SV_ values, in POPC liposomes (red) and PC/PG liposomes at 3:1 (green) and 3:2 (blue) molar ratios for iodide quenching of NBD-labelled slide helix mutants of KirBac1.1 are shown. Data for the inactive state (i.e. in POPC membranes) were adapted from [22]. See Materials and Methods and text for other detail

To understand the slide helix location in PG-containing membranes, we probed water accessibility using collisional quenching of NBD fluorescence by potassium iodide (KI) as the aqueous quencher [24,31,48]. Figure 5A shows the representative results for quenching of S46-NBD in POPC, PC/25 mol% PG, PC/40 mol% PG membranes as Stern-Volmer plots. The slope of such a plot (*i.e*. the Stern-Volmer constant, K_SV_) is related to the degree of exposure of the NBD group to water. However, it is intrinsically dependent on the fluorescence lifetime. Therefore, the bimolecular quenching constant (k_q_), which takes into account the differences in lifetimes and offers more precise information on the degree of water exposure, was calculated using k_q_ = K_SV_/τ, where τ is the fluorescence lifetime in the absence of quencher [24]. Figure 5B shows the calculated k_q_ values for the slide helix residues in both functional states. As previously reported [22], the k_q_ values of the slide helix in zwitterionic POPC membranes are in the range o f ~0.7 to 2 M^−1^ ns^−1^. In PC/PG membranes, reduced k_q_ values of the slide helix are obtained (~0.5 to 1.3 M^−1^ ns^−1^ and ~0.8 to 1.0 M^−1^ ns^−1^ in 25 and 40 mol% PG-containing membranes, respectively), which suggests that the water accessibility is significantly decreased near the slide helix residues. Considering the k_q_ value of ~8 M^−1^ ns^−1^ for the complete exposure of NBD to aqueous medium [24,48], these values clearly suggest that the slide helix residues are highly shielded from the aqueous exposure. This is in overall agreement with the pronounced increase in lifetimes of the NBD-labelled slide helix residues in PC/PG membranes (see Figure 3, C and D). Although the water accessibility is decreased for the slide helix in active conformation of KirBac1.1 (in anionic membranes) compared with inactive conformation (in POPC membranes), the effects of increasing PG content are not linear. Overall, these results clearly point out a change in reorganization of the slide helix in PG-containing membranes.

### Hydration dynamics of the slide helix in the active/open conformation of KirBac1.1

Intrinsic relation of protein dynamics to hydrating solvent molecules and slow solvation has been widely documented [49,50]. Further, hydration dynamics has been shown to play crucial roles in lipid-protein interactions [22,29–31,46,47] mediating ion channel functional states [20,51] and ion channel selectivity [52]. Red edge excitation shift (REES) is a well-established fluorescence approach, which provides novel insights on the relative rates of water relaxation dynamics in complex biological systems and is sensitive to changes in local hydration dynamics (see [53–55] for reviews). Further, the sensitivity of REES to changes in local hydration dynamics has been widely used as a powerful tool to probe the presence of restricted/bound water molecules, side-chain rearrangements in a protein core and protein conformational substates [55].

**Figure 6:**
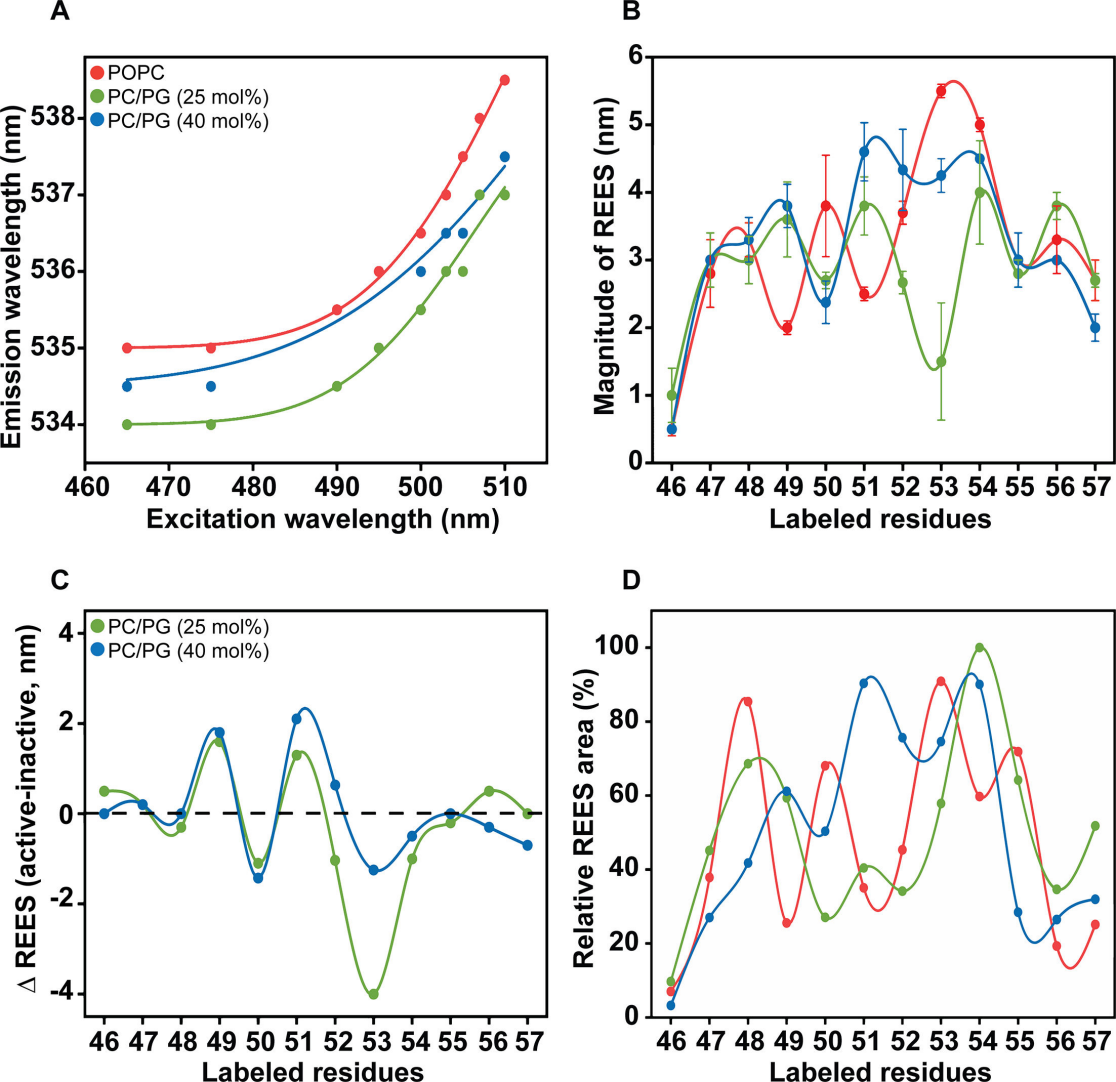
Anionic lipid induced changes in hydration dynamics. (**A**) Effect of changing excitation wavelength on the wavelength of maximum emission for D50-NBD in POPC liposomes (red) and PC/PG liposomes at 3:1 (green) and 3:2 (blue) molar ratios is shown. The solid lines are the fit to Eq. 6. (**B**) The magnitude of REES for the NBD-labelled slide helix residues in above-mentioned membranes is represented as mean ± SE of three independent measurements. (**C**) The change in hydration dynamics of the slide helix is shown as ΔREES (i.e. differences in the magnitude of REES in active and inactive states) in the presence of 25 (green) and 40 mol% (blue) PG lipid. (**D**) The relative area was calculated by fitting the REES data of the slide helix mutants using Eq. 6. The color legends for (**B**) and (**D**) are same as in (**A**). The joining lines in (**B**), (**C**), and (**D**) are provided merely as viewing guides. See Materials and Methods for other details. Data for the inactive state (*i.e*. in POPC membranes) were adapted from [22].

REES is operationally defined as the shift in the fluorescence emission maximum towards higher wavelengths caused by a shift in the excitation wavelength towards the red edge of the absorption band [54,55]. Figure 6A shows the representative data for change in emission maximum as a function of changing excitation wavelength from 465 to 510 nm for the D50-NBD residue of the slide helix in POPC and PC/PG membranes. The magnitude of REES, which is the total shift in emission maximum upon changing the excitation wavelength, is shown in Figure 6B. Irrespective of the membrane composition, all the slide helix residues, barring S46-NBD, show substantial REES suggesting the slow solvent relaxation associated with the presence of restricted/bound water molecules in the nanosecond time scale [53–55]. Interestingly, the periodic pattern of the REES values for the slide helix residues observed in POPC membranes is considerably phase shifted in PG-containing membranes. It should be noted that the fluorescence emission maximum for residues 50 to 52 is very similar in both POPC and PC/PG membranes (Figure 2A), yet the magnitude of REES changes from 2.5 to 4.5 nm in these membranes (Figure 6B). This clearly indicates that the observed differential hydration dynamics is not solely due to changes in the local polarity around the slide helix.

Compared with the hydration dynamics of the slide helix in POPC membranes, ΔREES values, i.e. the difference between PC/PG and POPC membranes, show that the presence of anionic lipid POPG significantly alters the hydration dynamics of the slide helix, especially for the residues 49 to 53 (Figure 6C). However, the N- and C- terminal ends of the slide helix do not show any appreciable changes. Interestingly, in the presence of varying amounts of PG lipid in the membrane, the pattern of changes in hydration dynamics of the slide helix is similar at the N-terminal half and quite different at the C-terminal half (Figure 6C). This pattern of two halves of the slide helix showing differential behaviour is somewhat similar to the differences in rotational correlation times (τ_c_) in PG-containing membranes (see Figure 4C). These results clearly indicate that the environmental restriction by the relaxing water molecules is significantly altered around the central part of the slide helix upon PG binding to the channel and could be attributed to lipid-driven reorganization of the slide helix in the active state of the channel.

Since increased magnitude of REES indicates the restricted environment around the fluorophore and increased anisotropy indicates the high molecular restriction, we directly compared ΔREES and Δτ_c_ values to get insights on the slide helix residues that undergo restricted mobility in the active state of the channel due to PG binding. Despite the environmental restriction around the slide helix residues being much reduced or not altered compared with POPC membranes, the Δτ_c_ values are significantly higher for residues 46, 50, 53, 55 and 57 when 40 mol% PG is incorporated in the membrane (Supplementary Figure S3). These results strongly support that the altered slide helix dynamics in the active/open state of KirBac1.1 is due to PG binding in the cationic binding site [16] at the cytoplasmic side of the membrane, and not merely due to solvent-induced environmental restriction.

Although the magnitude of REES is a very good qualitative measure to monitor relative solvent relaxation dynamics, the area extracted from the distinct curvature of REES data using the Gaussian probability distribution can effectively be used to probe changes in the equilibrium of protein conformational states [55,56]. We fitted our REES data with Eq. 6 to extract the relative area of the distribution for the NBD-labelled slide helix residues in inactive and active conformations and are shown in Figure 6D. Except for the N- and C-terminal residues of the slide helix, the relative area for all other residues is changed considerably in PG-containing membranes; yet, there is no correlation with the PG content. This suggests that the discrete conformational substates based on the slow hydration dynamics in the nanosecond time scale are significantly altered, and the restricted water–protein interactions are heterogeneous throughout the slide helix in membranes. We speculate that water molecules could act as a structural component in KirBac1.1 gating, similar to what has been shown for KcsA [51]. Taken together, our results are strongly in favour of structural reorganization of the slide helix at the interfacial region of anionic membranes.

### Conformational heterogeneity of the slide helix in the active state

**Figure 7:**
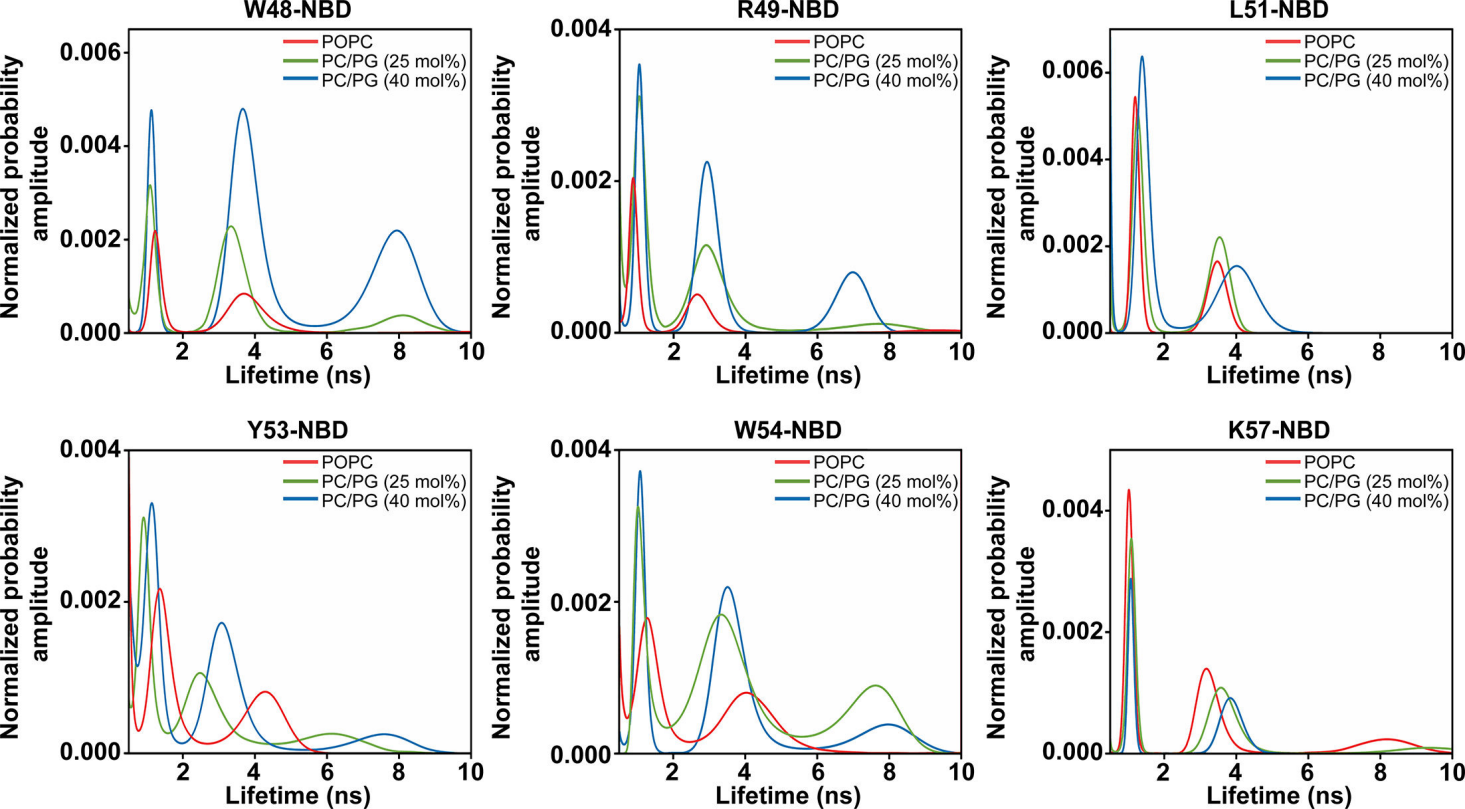
Conformational heterogeneity of the KirBac1.1. slide helix in membranes. MEM fluorescence lifetime distributions for the indicated NBD-labelled slide helix residues in POPC liposomes and PC/PG liposomes with varied PG content are shown. The normalized probability amplitudes are plotted against their corresponding lifetime on a linear scale. Data for Y53-NBD in the inactive state (i.e. in POPC membranes) were adapted from [22]. See Materials and Methods for details.

Our REES results suggested a change in conformational heterogeneity of the slide helix when KirBac1.1 undergoes PG-induced structural transition from inactive to active conformation. To check this possibility, we performed lifetime distribution analysis of the NBD-labelled slide helix mutants utilizing the maximum entropy method (MEM). Fluorescence decay analysis by MEM resolves the lifetime components in a model-independent manner [24,57], and importantly, it gives an ultrafast snapshot of the protein population distribution [58]. This powerful MEM approach has recently been utilized in KvAP voltage sensor [31,32] MgtE homologs [46,47], and KirBac1.1 [22] to monitor conformational heterogeneity. Figure 7 shows representative MEM lifetime distribution profiles of NBD-labelled slide helix residues 48, 49, 51, 53, 54 and 57 in POPC and PC/PG membranes. Basically, all lifetime distribution profiles contain multiple peaks, which is not surprising considering the complex nature of lipid-protein interactions in membranes. Having said that, more number of peaks are observed for most of the slide helix residues in PG-containing membranes compared with POPC membranes, demonstrating PG-driven altered conformational heterogeneity, which is in agreement with previous results. Interestingly, the conformational heterogeneity appears to be decreased from N- to C-terminal of the slide helix, which could be due to the change in location of the slide helix at the membrane interface (see below).

### Membrane penetration depths of the slide helix in close/inactive vs open/active state

**Figure 8:**
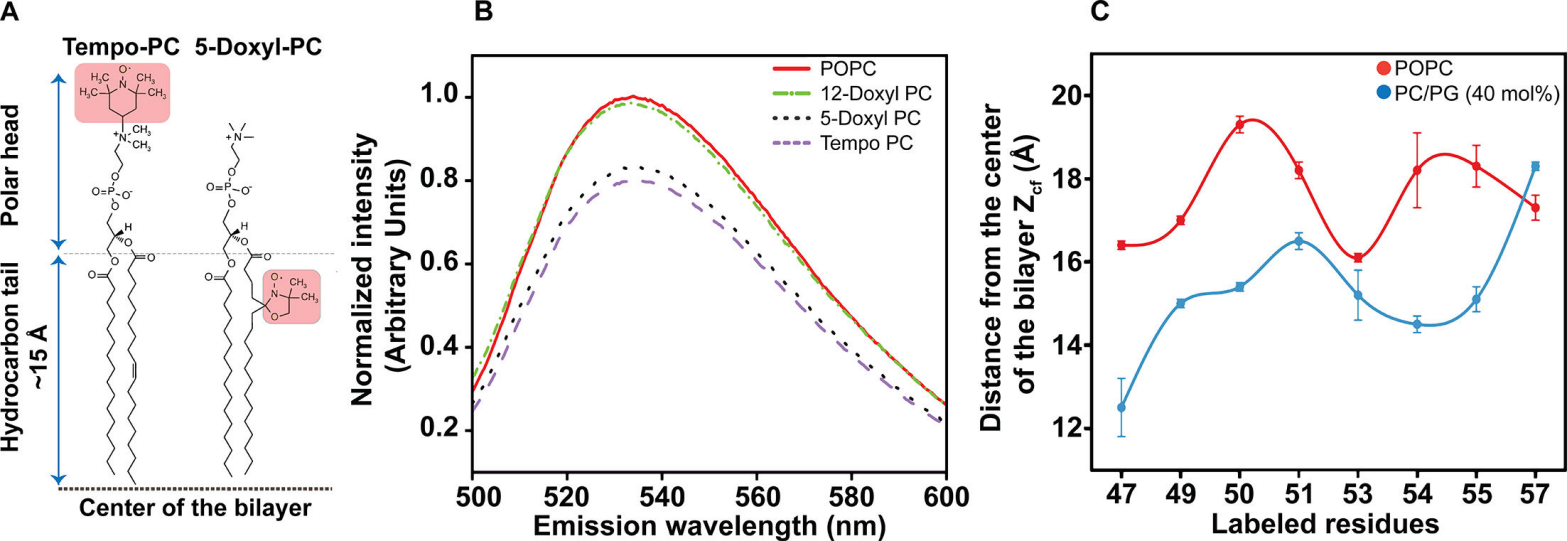
Membrane penetration depths of NBD-labelled slide helix residues in different functional states of KirBac1.1. (**A**) The chemical structures of Tempo-PC and 5-doxyl-PC and their positions from the center of the bilayer is shown. The headgroup labeling Tempo and the acyl chain labeling doxyl group are highlighted. (**B**) Representative fluorescence emission spectra of V47-NBD reconstituted in POPC liposomes in the absence (red) and presence of nitroxide quenchers, Tempo-PC (purple, dashed line), 5-doxyl PC (black, dotted line) and 12-doxyl PC (green, dot-dashed line) are shown. (**C**) The average distance measured for the NBD-labelled slide helix residues from the bilayer center in POPC (red) and PC/PG membranes containing 40 mol% PG (blue) is shown. The protein-to-lipid molar ratio was 1:100, and the concentration of protein used was 1.6 μM in all cases. The excitation wavelength was 465 nm and the intensity was monitored at respective emission maximum. The values represent mean ± SE of three independent measurements. See materials and methods for details.

Membrane penetration depth represents an important parameter in the study of the structure and organization of membranes and membrane proteins (see [59,60] for reviews). Further, fluorescence quenching-based distance measurements have been widely utilized to get crucial insights on the conformational changes in membrane peptides/proteins, particularly for the NBD-labelled systems [29,60–62]. To gain a better understanding of the PG-induced change in the organization and conformation of the slide helix in membranes, we measured the ‘average’ membrane penetration depths of the NBD group for some of the NBD-labelled slide helix residues using the parallax method [59,60,63]. Representative quenching of NBD fluorescence of 47-NBD of the slide helix by nitroxide quenchers Tempo-PC, 5-Doxyl PC and 12-Doxyl PC is shown in Figure 8B. In our case, Tempo-PC and 5-Doxyl-PC (Figure 8A) are the best quenching pair in all the mutants studied (see Supplementary Table S1).

The measured ‘average’ distances for the NBD-labelled slide helix residues in POPC and PC/PG membranes are shown in Figure 8C. The membrane penetration depths of the NBD-labelled slide helix in the inactive conformation of the channel (*i.e*. in POPC membranes) are within the range of 16–19 Å from the bilayer centre, which indicates that the slide helix is located at the shallow interfacial region of the POPC membrane. In contrast, the slide helix residues in the active conformation of KirBac1.1 (*i.e*. in PC/PG membranes) have increased membrane penetration depths, which is in the range of 12–18 Å. These results clearly show that the slide helix is located at the membrane interface in both functional states of the channel, which is in agreement with the amphipathic nature of the slide helix. Importantly, the slide helix partitions deeper at the membrane interface (~3 Å) in the PG-induced active conformation of the channel. Overall, our results demonstrate that the organization of the slide helix is functional state-dependent. Since the periodic nature of the depth profiles of the slide helix is phase shifted at the C-terminal half, it is possible that, during the PG activation of the channel, the slide helix might undergo tilt/bend and rotation due to PG binding-induced conformational changes.

## Discussion

Biological membranes, which are the site for many vital physiological functions, are dynamic and complex structures that actively regulate the organization and function of membrane proteins rather than merely serving as passive barriers [64]. In this process, lipid-protein interactions are central in stringently regulating the activity of membrane proteins, signal transduction and host-pathogen interactions [65–70]. Various classes of lipids in general, and anionic phospholipids in particular, act as annular or non-annular lipids and affect the function of membrane proteins. While annular lipids interact weakly with the membrane protein and are in rapid exchange with bulk lipids, the non-annular lipids bind to protein with specific high-affinity [65,66,71]. Lipid nanoenvironment, therefore, modulates the functional equilibrium of membrane proteins either by stereospecific, high-affinity interactions or by altering the bulk membrane properties or a combination of both [66,72]. The modulation of the function of membrane proteins, particularly the ion channels, by direct interaction with membrane lipids continues to be an emerging theme [73–75].

It is well documented that anionic lipids regulate the activity of Kir channels [14,73,74,76], which belong to a large superfamily of K^+^ ion channels and are important drug targets for several diseases [77,78]. Being a prokaryotic homolog of Kir channels, KirBac1.1 has been studied as a model system to understand the gating mechanism of Kir channels [8–17]. Like its eukaryotic counterparts, the channel activity of KirBac1.1 is regulated by negatively charged lipids, particularly PG lipid [14–16,76], which is a major component of bacterial membranes [79]. Among the negatively charged headgroup moieties, glycerol has the distinct water-mimicking structural properties that confer an increased solvation and hydrogen bonding ability [80].

The amphipathic slide helix of KirBac1.1, which resides towards the cytoplasmic interface near the helix bundle crossing, has been shown to be an important structural component in KirBac1.1 gating. For instance, it has been proposed that the slide helix controls channel gating by forming a link between pore domain and the cytoplasmic domain [17], and lipid tethering of the slide helix differentially modulates the channel activity [9]. Recently, the slide helix motion is hypothesized to increase the accessibility of PG binding sites in adjacent subunits, thereby achieving the mechanism of cooperativity during lipid-dependent gating [16]. In particular, the cation-binding pocket comprising R49 (part of slide helix), R151 and R153 has been proposed to interact with negatively charged PG lipid to activate the channel [14]. Considering the fact that the high-resolution structural snapshot of the active conformation of KirBac1.1 is not yet available and the nature of slide helix motion is not properly understood, probing the structural dynamics of functionally important slide helix in inactive/close and active/open states assumes significance and is the focus of this work.

Here, we have used a differential lipid reconstitution approach using the zwitterionic lipids (POPC) and a mixture of zwitterionic and anionic lipids (POPC and POPG) to stabilize the closed/inactive and open/active conformations of KirBac1.1, respectively, and utilized site-directed NBD fluorescence. Using ACMA-based liposome flux assay, we show that all the single-cysteine mutants of KirBac1.1 (residues 46 to 57) remain functionally active and display PG lipid-driven gating of the channel. Although negatively charged PG lipid activates the channel, increasing the PG content from 25 to 40 mol% does not have any linear dependency both on the activation and the K^+^ flux rate (see Figure 2, E and F). It should be noted that, utilizing the Rb^+^ flux assay, it has previously been shown that the single cysteine mutants of R49 and K57 of the slide helix show negligible channel activity in 3:1 POPE/POPG membranes [9]. In our case, the channel activity of the R49C mutant is similar to wildtype, and K57C is functionally active despite showing reduced activity compared with wildtype (see Figure 2E). Since the arginine triad (R49/R151/R153) has been shown to be important for activating the channel [14], we therefore propose that mutating R49 alone may not be sufficient to make the channel inactive and the concerted role of these ‘gating’ arginines might be required. Similarly, the slide helix W48C mutant displays K^+^ transport activity much like the wildtype channel in PG-containing membranes, suggesting that the interesting observation of W60 at TM1 moving towards W48 as a result of PG binding [16,81] may not be an absolute requirement for PG-induced lipid-dependent gating.

Conflicting models have emerged for the location of slide helix in KirBac1.1. Based on the crystal structure, the slide helix is predicted to lie parallel to the membrane in the vicinity of phospholipid headgroups [17]. Another model considers the slide helix as a part of cytoplasmic domain and controls the channel activity by lipid tethering [9]. Our results, utilizing the site-directed steady-state and time-resolved NBD fluorescence approaches, reveal that the slide helix is well-partitioned at the membrane interface in both inactive and active conformations of KirBac1.1. Membrane interface constitutes ~50% of the membrane bilayer thickness (~15 Å per leaflet) and is a chemically heterogeneous region comprising lipid headgroups, water and portions of the acyl chain, and is characterized by unique motional and dielectric characteristics distinct from the bulk aqueous phase and the hydrocarbon-like interior of the membrane [82]. Considering the amphipathic nature of the slide helix and its hydrophobic moment (0.416) and hydrophobicity (0.712) values [22], the membrane interfacial localization of the slide helix in both functional states is expected.

**Figure 9:**
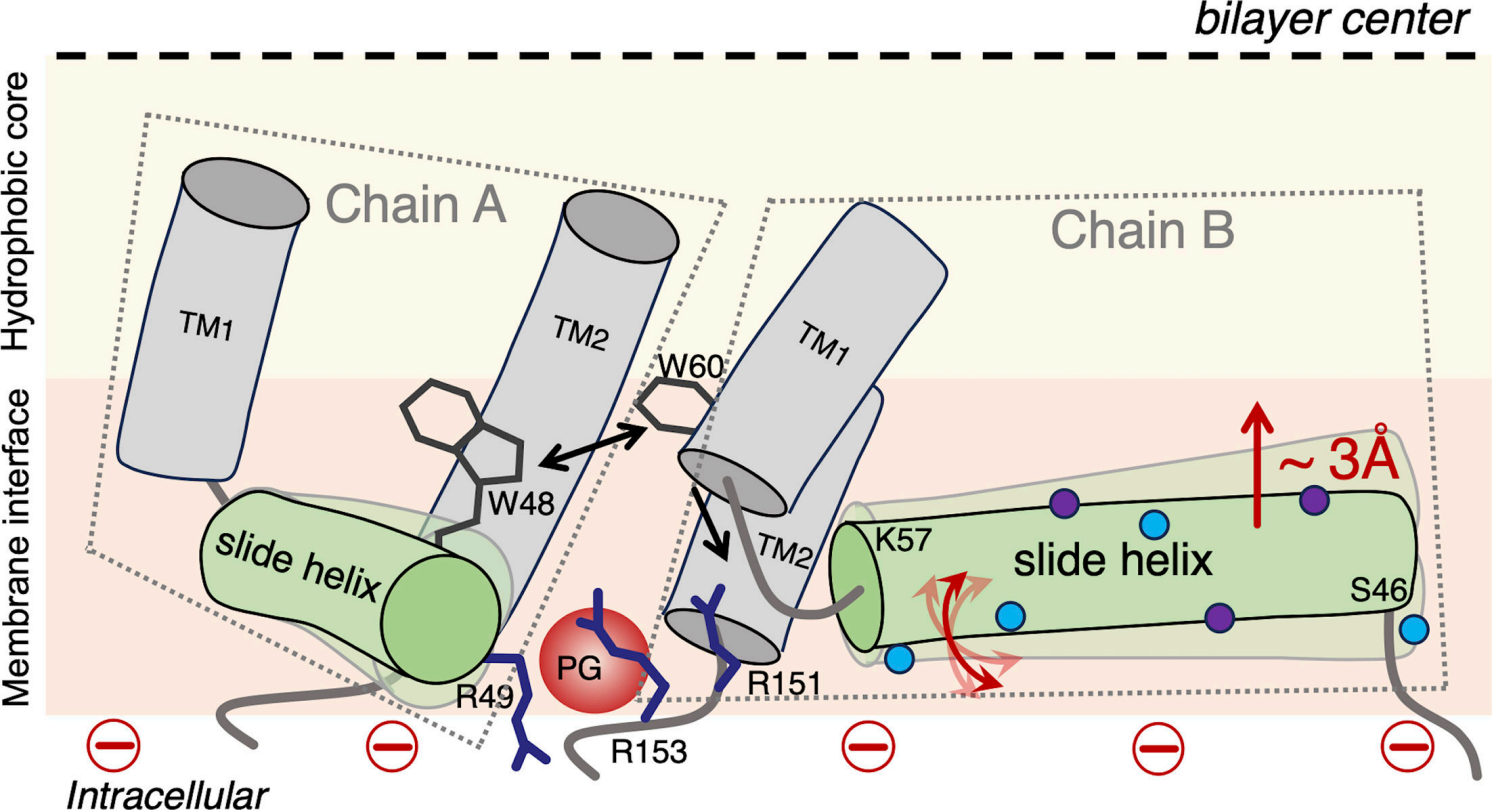
Reorganization of the slide helix in the active/open conformation of KirBac1.1. A schematic representation highlighting the structural dynamics changes in the slide helix upon PG-dependent gating is shown. Only two subunits (chains A and B) are shown for clarity to depict the PG (denoted as red sphere) interaction at the cationic binding pocket, which is mainly comprised of three arginine residues (represented as blue sticks) - R49 (from Chain A), R151 and R153 (Chain B). The dotted quadrilaterals are used to demarcate chains A and B. The cytoplasmic leaflet of the membrane bilayer is shown, and the dashed black line depicts the bilayer center. The presence of the negatively charged PG lipid in membranes will result in increased membrane surface charge (shown as red minus signs). Only a part of TM1 and TM2 helices from both chains are shown. The amphipathic slide helix (green cylinder) is shown with the approximate positions of the N-terminal (**S46**) and C-terminal (**K57**) residues. Compared with its inactive conformation in zwitterionic POPC membranes, the slide helix is partitioned at deeper interfacial region (denoted by a straight red arrow) by ~3 Å, which might induce conformational changes in TM1 (black down arrow) to bring W48 (from Chain A) and W60 (from Chain B) relatively closer, depicted as double-headed black arrow. Between the active and inactive states of the channel, the middle part of the slide helix undergoes differential hydration dynamics, which is denoted by small spheres that represent the free/bulk (cyan) and restricted/bound (purple) water molecules. The C-terminal of the slide helix experiences dynamic fluctuations (denoted by double-headed curved red arrows), whereas the rotational dynamics of the N-terminal half is not only highly restricted but also has increased conformational heterogeneity, which is denoted by multiple copies of the slide helix. See text for details.

Based on our results, we propose a model (Figure 9) that highlights the key differences in the organization and dynamics of the slide helix when KirBac1.1 undergoes structural transition from inactive/close (POPC membranes) to active/open (PC/PG membranes). Due to PG binding in the cationic binding pocket of KirBac1.1, the dynamics exhibited by the slide helix is segmental in nature, *i.e*. the N-terminal half undergoes restricted mobility at high concentration of PG whereas the C-terminal half experiences dynamic fluctuations (Figure 4C). This is accompanied by the reduced water accessibility (Figure 5B) and differential hydration dynamics (Figure 6B) around the slide helix, which could possibly be due to lipid-driven reorganization of the slide helix in the active state of the channel. Considering the functional correlations of hydration and conformational dynamics in inactivating and conductive conformations of K^+^ channels [20,21,51], our results showing the presence of restricted/bound water molecules in the immediate vicinity of the slide helix might also be relevant for lipid-dependent gating. Comparison of differences in solvent relaxation dynamics and the rotational correlation times between two functional states of KirBac1.1 (see Supplementary Figure S3) strongly indicates that the altered slide helix dynamics in the active/open state of KirBac1.1 is due to conformational changes associated with the PG binding, *i.e*. reorganization of the slide helix upon gating transition. This is in agreement with lifetime distribution analysis, which shows that the conformational heterogeneity appears to be decreased from N- to C-terminal of the slide helix (Figure 7). Importantly, although the slide helix resides at the membrane interface in both functional states, our membrane penetration depth measurements show that the slide helix partitions ~3 Å deeper in the membrane interface in the active state of the channel (Figure 8), confirming the reorganization of the slide helix, which might include tilt/bend and rotation, during PG-induced activation. It is possible that this PG-induced reorganization of the slide helix might induce conformational changes in TM1 to bring W48 and W60 from adjacent subunits relatively closer as shown previously [16,81]. The fact that the observed changes in structural dynamics in anionic membranes do not change proportionally with respect to changing the PG content in membranes probably suggests a complex lipid–protein interactions, which might involve both the contributions from the non-annular lipid interactions as well as the change in membrane bilayer properties due to increase in membrane surface charge. Overall, our results bring out the importance of lipid-protein interactions and are relevant for understanding the lipid-dependent gating of membrane transport proteins in general, and Kir channels in particular. In view of the emerging field of ‘membrane lipid therapy’ to pharmacologically regulate membrane lipid composition for the treatment of diseases [83], our results assume significance.

## Materials and methods

### Materials

*E. coli* BL21(DE3) strain was purchased from Agilent (Santa Clara, CA). n-decyl-β-D-maltopyranoside (DM) was obtained from Anatrace (Maumee, OH). PMSF (phenylmethylsulfonyl fluoride) was obtained from GoldBio (St. Louis, MO). 1-palmitoyl-2-oleoyl-sn-glycero-3-phosphocholine (POPC), 1-palmitoyl-2-oleoyl-sn-glycero-3-phospho-1′-rac-glycerol (POPG), 1-palmitoyl-2-oleoyl-sn-glycero-3-phospho(tempo)choline (Tempo-PC), 1-palmitoyl-2-stearoyl-(5-doxyl)-sn-glycero-3-phosphocholine (5-doxyl-PC) and 1-palmitoyl-2-stearoyl-(12-doxyl)-sn-glycero-3-phosphocholine (12-doxyl-PC) were obtained from Avanti Polar Lipids (Alabaster, AL). IANBD amide (N, N′- dimethyl-N-(iodoacetyl)-N′-(7-nitrobenz-2-oxa-1,3-diazol-4-yl) and ACMA (9-amino-6-chloro-2-methoxyacridine) were purchased from Invitrogen (Carlsbad, CA). All other chemicals used were of the highest purity available from either Merck (Kenilworth, NJ) or Amresco (Radnor, PA).

### Mutagenesis, channel expression and purification

The gene encoding the full-length wildtype KirBac1.1 from *Burkholderia pseudomallei* was cloned into a pQE60 vector with a C-terminal 6x-His-tag (Qiagen, Hilden, Germany). Single-cysteine mutants were generated for residues 46 to 57 corresponding to the slide helix of KirBac1.1 using KOD-Plus-High fidelity DNA polymerase kit. The mutations were confirmed by DNA Sanger sequencing. The wildtype and single-cysteine mutants of KirBac1.1 were expressed and purified as described previously [84]. The concentration of the protein was checked in a DS-11+microvolume spectrophotometer (DeNovix, Wilmington, DE). To analyse whether the channel is folded properly, the purified protein (~148 kDa, tetramer) was applied onto a Superdex 200 increase 10/300 GL (GE Healthcare, Chicago, IL) size-exclusion column equilibrated with 50 mM Tris, 150 mM KCl, 5 mM DM (pH 8.0) buffer.

### Site-specific fluorescence labelling and membrane reconstitution

The purified single cysteine mutants of KirBac1.1 were fluorescently labelled using IANBD amide, which is a thiol-reactive environment sensitive fluorescent probe, as described earlier [24]. The labelled mutants of KirBac1.1 were reconstituted at a lipid to protein molar ratio of 100:1 in POPC, POPC/POPG (3:1 mol/mol) and POPC/POPG (3:2 mol/mol) liposomes. We have used PG content of 25 mol% and 40 mol% in POPC/POPG (3:1) and POPC/POPC (3:2) liposomes, respectively, in line with the previous studies [14–16,84]. The membrane reconstitution of labelled KirBac1.1. mutants was carried out as follows: Briefly, lipids in desired ratios in chloroform were dried under a stream of nitrogen while being warmed gently (~35°C). After the lipids were dried further under a high vacuum for at least 3 hr, they were hydrated (swelled) by adding 1 ml of 50 mM Tris, 150 mM KCl (pH 8.0) buffer and vortexed vigorously for 3 min to disperse the lipids and sonicated to clarity. Labelled protein was then added to give a molar ratio of 100:1 lipid: KirBac1.1. The sample was left at room temperature for 30 minutes on a rotator and 200 mg of pre-washed biobeads (SM-2, Bio-Rad, Hercules, CA) were then added and the mixture was incubated on a rotator overnight at 4°C to remove the detergent. The biobeads were removed by filtering using a Bio-Rad 5 ml column filter before use.

### CD measurements

CD measurements were carried out at room temperature in a Jasco J-1100 spectropolarimeter purged with a nitrogen flow of 5 l/min. Wildtype and NBD-labelled single-cysteine mutants were measured at a concentration of 5 μM in POPC and POPC/POPG (at 3:1 and 3:2 molar ratios) liposomes at a lipid:protein ratio of 100:1 (mol/mol) to obtain a good signal-to-noise ratio. The buffer used for making the liposomes was 50 mM Tris, 150 mM KCl, pH 8.0. The spectra were scanned with a quartz optical cuvette with a pathlength of 0.1 cm. All spectra were recorded with a bandwidth of 1 nm and integration time of 0.5 s with a scan rate of 50 nm/min. Each spectrum is the average of 10 scans. All spectra were appropriately blank subtracted and smoothed so as to ensure that the overall shape of the spectra remains unaltered. The ellipticity data obtained in millidegree were converted to molar ellipticity ([*θ*]) by using the following equation:

Eq. 1 $$\left[\theta \right]=\frac{\theta_{obs}}{10Cl}$$

where *θ_obs_* is the observed ellipticity in millidegree, *C* is the concentration in mol/L and *l* is the pathlength in cm.

### Cuvette-based liposomal flux assay for potassium transport

The fluorescence assay for K^+^ transport using the pH gradient-sensitive fluorophore ACMA was carried out as described previously [14,33,34]. KirBac1.1 was reconstituted in POPC and POPC/POPG (3:1 mol/mol and 3:2 mol/mol) liposomes as described above with few modifications. Briefly, the dried lipids (1280 nmoles) were hydrated (swelled) by adding internal buffer (1 ml of 20 mM HEPES, 150 mM KCl, pH 8.0), vortexed vigorously for 2 min to disperse the lipids and sonicated to clarity. Proteoliposomes were made by reconstituting 3.8 μM of protein at a lipid-to-protein molar ratio of ~300:1. 100 μl of proteoliposomes (*i.e*. membrane reconstituted wildtype and the single-cysteine mutants of the slide helix of KirBac1.1) and 1 μM ACMA were added to a cuvette containing 2 ml of potassium-free buffer (150 mM NMDG-Cl, 20 mM HEPES, pH 8.0), which was used to generate an outward potassium gradient, and incubated in dark for 10 min. Continuous recording of fluorescence emission intensity of ACMA at 480 nm while exciting at 410 nm, in a 3 ml quartz cuvette with constant stirring, was measured at an interval of 5 s at room temperature in a PTI Quantamaster (Horiba) spectrofluorometer using a nominal bandpass of 2.5 nm by 2.5 nm. Outward K^+^ flux was initiated by adding 3.2 μM CCCP after making sure the intensity baseline was flat and 100 nM valinomycin (Val) was added after 600 s to estimate the total K^+^ flux. Experiments with protein-free liposomes served as control. All data were normalized using the following equation after subtracting the fluorescence intensity obtained from control liposomes:

   Eq. 2 $$F_{N}=\left(F_{c}-F\right)/\left(F_{c}-F_{v}\right)$$

where *F*_*N*_ stands for normalized fluorescence, *F* is the measured fluorescence of the sample before addition of valinomycin (595 s), *F*_*v*_ is the fluorescence after adding the valinomycin, and *F*_*c*_ is the initial fluorescence intensity before adding the CCCP (60 s). Relative K^+^ flux rate was calculated by fitting the normalized fluorescence quenching (decay) curve (i.e. from the point of addition of CCCP to the point before addition of valinomycin) to the following monoexponential decay function:

     Eq. 3 $$F_{N}\left(t\right)=F_{o}*exp^{-t/\tau }+c$$

where *F*_*N*_(*t*) is for normalized fluorescence at time *t*, *F*_*o*_ is the fluorescence intensity at the point of addition of CCCP, *t* is read time in seconds, 1/τ is the decay rate of fluorescence signal in seconds, and *c* is a constant.

### Steady-state fluorescence measurements

Steady-state fluorescence measurements were performed with PTI Quantamaster (Horiba) spectrofluorometer using 1 cm path length quartz cuvettes. Excitation and emission slits with a nominal bandpass of 5 nm were used for all measurements. Background intensities were appropriately subtracted from each sample spectrum to cancel out any contribution due to the solvent Raman peak and other scattering artefacts. Fluorescence emission spectra, at the excitation wavelength of 465 nm, were recorded for NBD-labelled slide helix residues of KirBac1.1 reconstituted in POPC and POPC/POPG (at 3:1 mol/mol and 3:2 mol/mol) membranes. Fluorescence anisotropy measurements were performed at room temperature using Horiba polarization accessory. Anisotropy values were calculated from the equation [24]:

      Eq. 4 $$r=\frac{I_{VV}-GI_{VH}}{I_{VV}+2GI_{VH}}$$

where, *I_VV_* and *I*_*VH*_ are the measured fluorescence intensities (after appropriate background subtraction) with the excitation polarizer vertically oriented and emission polarizer vertically and horizontally oriented, respectively. G is the grating correction factor and is the ratio of the efficiencies of the detection system for vertically and horizontally polarized light, and is equal to *I_HV_/I_HH_*. The apparent (average) rotational correlation times were calculated using Perrin’s equation [24]:

      Eq. 5 $$\tau_{c}=\frac{<\tau >r}{\left(r_{o}-r\right)}$$

where *r_o_* is the limiting anisotropy of NBD (0.354) [25], *r* is the steady-state anisotropy, and <*τ* > is the mean fluorescence lifetime.

REES measurements were done by measuring the emission maximum as a function of increasing excitation wavelength from 465 to 510 nm. The magnitude of REES represents the total shift in emission maximum upon the indicated change in the excitation wavelength [24]. The REES data were fitted by a Gaussian probability distribution of the form [58]:

   Eq. 6 $$f\left(x\right)=R_{0}+\frac{A\sqrt{2/\pi }}{w}exp\left(-2{\left(\frac{x-m}{w}\right)}^{2}\right)$$

where *A* is the area, *w* is the full width at half-maximum (FWHM), *m* is the midpoint and *R*_0_ is the y-intercept and *m* is the excitation wavelength that gives the largest change in the emission peak wavelength.

### Time-resolved fluorescence measurements

Fluorescence lifetimes were calculated from time-resolved fluorescence intensity decays using HORIBA Fluoromax-3 in time-correlated single-photon counting (TCSPC) mode with a picosecond pulsed 465 nm Nano-LED as the light source. Lamp profiles were measured at the excitation wavelength using Ludox (colloidal silica) as the scatterer. To optimize the signal/noise ratio, 10,000 photon counts were collected in the peak channel. All experiments were performed with a bandpass of 5–8 nm. Fluorescence intensity decay curves so obtained were deconvoluted with the instrument response function and analysed as a sum of exponential terms:

     Eq. 7 $$F\left(t\right)=\sum_{i}\alpha_{i}exp\left(-t/\tau_{i}\right)$$

where *F(t*) is the fluorescence intensity at time *t* and *α*i is a pre-exponential factor representing the fractional contribution to the time-resolved decay of the component with a lifetime *τ*_i_ such that Σ_i_ *α*_i_ = 1. Mean (average) lifetimes <τ > for triexponential decays of fluorescence were calculated from the decay times and preexponential factors using the following equation [24]:

     Eq. 8 $$<\tau >=\frac{\alpha_{\text{1}}\tau_{\text{1}}^{2}+\alpha_{\text{2}}\tau_{\text{2}}^{2}+\alpha_{\text{3}}\tau_{\text{3}}^{2}}{\alpha_{\text{1}}\tau_{\text{1}}+\alpha_{\text{2}}\tau_{\text{2}}+\alpha_{\text{3}}\tau_{\text{3}}}$$

The fluorescence decay data analysis by maximum entropy method (MEM) was carried out as described in detail previously [24]. MEM represents a convenient, robust, model-free and realistic approach to data analysis [57]. All MEM fits were performed on a standard PC using the open access AnalyseDistribution MATLAB code (see ref [57] for further details).

### Fluorescence quenching measurements

Collisional quenching experiments of NBD fluorescence in POPC and POPC/POPG liposomes were carried out as described previously [24]. Briefly, the fluorescence intensity of NBD-labelled KirBac1.1 slide helix mutants in POPC and PC/PG liposomes was measured by sequential addition of freshly prepared stock solution (2.5 M) of KI having 5 mM sodium thiosulphate pentahydrate (Na_2_S_2_O_3_.5H_2_O) in water to each sample followed by incubation for 2 min in the sample compartment in the dark (shutters closed). The excitation wavelength used was 465 nm and emission was monitored at the respective emission maximum. The quenching data were corrected for dilution and inner filter effects and were subsequently analysed by fitting to the Stern-Volmer equation as described previously [24].

### Depth measurements using the parallax method

For depth measurements of the NBD-labelled mutants of KirBac1.1 in POPC and POPC/POPG (3:2 mol/mol) membranes, three sets of samples were used in which 160 nmol of total lipid was used containing 10 mol% spin-labelled phospholipids (Tempo-PC or 5-doxyl PC or 12-Doxyl PC) for each sample. The purified labelled protein was added to the preformed lipid vesicles at 100:1 lipid/KirBac1.1 and rotated at room temperature for 30 min followed by addition of 200 mg of prewashed biobeads to remove detergents and incubated overnight on a rotator at 4°C. The biobeads were removed by filtering using a Bio-Rad 5 ml column filter before use. The lipid composition of these samples was as follows: i) POPC (100 mol%); ii) POPC (90 mol%) and (Tempo-/5-Doxyl/12-Doxyl)-PC (10 mol%); iii) POPC/POPG (3:2 mol/mol) (100 mol%) and iv) POPC/POPG (3:2 mol/mol) (90 mol%) and (Tempo-/5-Doxyl/12-Doxyl)-PC (10 mol%), Triplicate samples were prepared in each case. Background samples lacking the fluorophore were prepared in all experiments, and their fluorescence intensity was subtracted from the respective sample fluorescence intensity. Samples were kept in the dark for 1 h before measuring fluorescence. Depths of the NBD-labelled slide helix residues were calculated using the best quenching pair (Tempo-PC and 5-Doxyl PC in our case) by the parallax method using the equation [63]:

  Eq. 9 $$Z_{cF}=L_{c1}+\left[\left\{\left(-1/\pi C\right)ln\left(F_{1}/F_{2}\right)–L_{21}^{2}\right]/2L_{21}\right\}$$

where *Z*_*cF*_ is the depth of the fluorophore from the center of the bilayer, *L*_*c1*_ the distance of the center of the bilayer from the shallow quencher (Tempo-PC in this case), *L*_*21*_ the difference in depth between the two quenchers (i.e. the transverse distance between the shallow and the deep quencher), and *C* the two-dimensional quencher concentration in the plane of the membrane (molecules/Å^2). Here *F*_1_/*F*_2_ is the ratio of *F*_1_/*F*_o_ and *F*_2_/*F*_o_ in which *F*_1_ and *F*_2_ are fluorescence intensities in the presence of the shallow (Tempo-PC) and deep quencher (5-Doxyl PC), respectively, both at the same quencher concentration *C*; *F*_o_ is the fluorescence intensity in the absence of any quencher.

## Supplementary material

Online supplementary material 1

## Data Availability

All data are contained within the manuscript.

## Open Access

This article has been published open access under our Subscribe to Open programme, made possible through the support of our subscribing institutions, learn more here: https://portlandpress.com/pages/open_access_options_and_prices#conditional

## Abbreviations

ACMA: 9-amino-6-chloro-2-methoxyacridine
CCCP: carbonyl cyanide m-chlorophenyl hydrazone
CD: circular dichroism
DM: n-decyl-β-D-maltopyranoside
5-Doxyl-PC: 1-palmitoyl-2-stearoyl-(5-doxyl)-sn-glycero-3-phosphocholine
12-Doxyl PC: 1-palmitoyl-2-stearoyl-(12-doxyl)-sn-glycero-3-phosphocholine
HEPES: hydroxyethylpiperazine ethane sulfonic acid
IPTG: isopropylthiogalactoside; MEM, maximum entropy method
NBD: 7-nitrobenz-2-oxa-1,3,-diazol-4-yl
NMDG-Cl: N-methyl-D-glucamine chloride
PDB: protein data bank
POPC: 1-palmitoyl-2-oleoyl-sn-glycero-3-phosphocholine
POPG: 1-palmitoyl-2-oleoyl-sn-glycero-3-phospho-(1′-rac-glycerol) (sodium salt)
REES: red edge excitation shift
SEC: size exclusion chromatography
TCSPC: time-correlated single photon counting
TM: transmembrane
Tempo-PC: 1-palmitoyl-2-oleoyl-sn-glycero-3-phospho(tempo)choline
