# Supplementary material for: Anionic lipids modulate the membrane localization and conformational dynamics of KirBac1.1 slide helix during lipid-dependent activation
Source: Biochem J. 2025 Sep 30;482(19):1393–413. doi: 10.1042/BCJ20253215 (PMC12599233; doi:10.1042/BCJ20253215)
Supplement: Online supplementary material 1 [file bcj-482-19-BCJ20253215-s001.docx]

**Supporting Information**

**Anionic lipids modulate the membrane localization and conformational dynamics of KirBac1.1 slide helix during lipid-dependent activation**

**Arpan Bysack^1,2^, Chandrima Jash^1^ and H. Raghuraman^1,2*^**

^1^Crystallography and Molecular Biology Division, Saha Institute of Nuclear Physics,

Kolkata, India

^2^Homi Bhabha National Institute, Training School Complex, Mumbai, India

Running title: *KirBac1.1 slide helix dynamics during lipid-dependent gating*

^*^Corresponding author.

Tel: +91-33-23375345, Extn: 1314

Fax: +91-33-23374637

E-mail: [h.raghuraman@saha.ac.in](mailto:h.raghuraman@saha.ac.in)

**List of Supporting Information files and Description**

**Table S1**: Fluorescence lifetimes of NBD-labeled slide helix mutants in membranes

**Table S2**: Efficiency of spin-labels in quenching the NBD fluorescence of NBD-labeled slide helix mutants of KirBac1.1 in different membranes

**Figure S1**: NBD-labeling does not affect the PG-dependent gating of KirBac1.1.

**Figure S2**: Amino acids surrounding slide helix residues.

**Figure S3**: Comparison of changes in hydration dynamics and rotational dynamics upon PG-induced gating.

**Table S1 Fluorescence lifetimes of NBD-labeled slide helix mutants in membranes^*^**

|  | α_1_ | τ_1_  (ns) | α_2_ | τ_2_  (ns) | α_3_ | τ_3_  (ns) | <τ>  (ns)^#^ | χ^2^ |
| --- | --- | --- | --- | --- | --- | --- | --- | --- |
| *POPC membranes* | | | | | | | |  |
| S46 | 0.39 | 1.76 | 0.39 | 4.53 | 0.23 | 0.53 | 3.6 | 1.17 |
| V47 | 0.37 | 1.53 | 0.40 | 4.37 | 0.23 | 0.22 | 3.6 | 0.99 |
| W48 | 0.35 | 1.52 | 0.30 | 4.89 | 0.35 | 0.21 | 3.9 | 1.04 |
| R49 | 0.14 | 3.76 | 0.28 | 1.09 | 0.58 | 0.21 | 2.4 | 1.06 |
| D50 | 0.36 | 1.04 | 0.24 | 3.88 | 0.41 | 0.17 | 2.9 | 1.08 |
| L51 | 0.37 | 1.14 | 0.26 | 3.58 | 0.37 | 0.12 | 2.7 | 1.13 |
| Y52 | 0.43 | 1.57 | 0.32 | 5.18 | 0.25 | 0.29 | 4.0 | 1.16 |
| Y53 | 0.40 | 1.26 | 0.29 | 4.23 | 0.31 | 0.21 | 3.2 | 1.14 |
| W54 | 0.37 | 1.64 | 0.30 | 5.04 | 0.33 | 0.41 | 3.8 | 1.09 |
| A55 | 0.34 | 1.58 | 0.23 | 5.10 | 0.43 | 0.40 | 3.6 | 1.15 |
| L56 | 0.31 | 1.49 | 0.25 | 5.18 | 0.44 | 0.35 | 3.9 | 1.11 |
| K57 | 0.31 | 1.33 | 0.19 | 4.18 | 0.50 | 0.42 | 2.8 | 1.02 |
| *PC/PG (3:1) membranes* | | | | | | | |  |
| S46 | 0.41 | 2.34 | 0.32 | 5.12 | 0.28 | 0.77 | 3.8 | 1.04 |
| V47 | 0.69 | 1.65 | 0.46 | 4.66 | 0.15 | 0.17 | 3.8 | 1.18 |
| W48 | 0.44 | 1.89 | 0.38 | 5.38 | 0.18 | 0.33 | 4.3 | 1.14 |
| R49 | 0.41 | 1.19 | 0.29 | 4.10 | 0.30 | 0.20 | 3.1 | 1.11 |
| D50 | 0.39 | 1.34 | 0.36 | 4.33 | 0.26 | 0.23 | 3.5 | 1.07 |
| L51 | 0.41 | 1.19 | 0.29 | 3.70 | 0.30 | 0.21 | 2.8 | 1.02 |
| Y52 | 0.32 | 1.65 | 0.38 | 5.58 | 0.30 | 0.15 | 4.7 | 1.08 |
| Y53 | 0.41 | 1.24 | 0.30 | 4.36 | 0.29 | 0.18 | 3.4 | 1.11 |
| W54 | 0.42 | 2.26 | 0.45 | 6.19 | 0.13 | 0.46 | 5.1 | 1.06 |
| A55 | 0.29 | 1.40 | 0.31 | 5.15 | 0.40 | 0.14 | 4.3 | 1.14 |
| L56 | 0.33 | 1.88 | 0.25 | 5.73 | 0.42 | 0.44 | 4.2 | 1.16 |
| K57 | 0.33 | 1.19 | 0.29 | 4.64 | 0.38 | 1.16 | 3.7 | 1.10 |
| *PC/PG (3:2) membranes* | | | | | | | |  |
| S46 | 0.39 | 1.94 | 0.38 | 4.79 | 0.23 | 0.65 | 3.7 | 1.07 |
| V47 | 0.37 | 1.37 | 0.41 | 4.30 | 0.22 | 0.16 | 3.6 | 1.09 |
| W48 | 0.34 | 2.45 | 0.56 | 5.61 | 0.10 | 0.28 | 4.9 | 1.18 |
| R49 | 0.38 | 1.68 | 0.36 | 4.31 | 0.27 | 0.26 | 3.4 | 1.18 |
| D50 | 0.32 | 1.18 | 0.29 | 4.14 | 0.40 | 0.15 | 3.3 | 1.10 |
| L51 | 0.43 | 1.29 | 0.32 | 3.95 | 0.25 | 0.25 | 3.1 | 1.10 |
| Y52 | 0.43 | 2.07 | 0.33 | 6.14 | 0.24 | 0.52 | 4.7 | 1.12 |
| Y53 | 0.37 | 1.75 | 0.27 | 4.88 | 0.36 | 0.52 | 3.5 | 1.18 |
| W54 | 0.36 | 1.75 | 0.46 | 5.30 | 0.18 | 0.24 | 4.5 | 1.20 |
| A55 | 0.36 | 1.25 | 0.29 | 4.72 | 0.34 | 0.17 | 3.8 | 1.06 |
| L56 | 0.34 | 1.70 | 0.28 | 5.57 | 0.37 | 0.39 | 4.3 | 1.08 |
| K57 | 0.24 | 1.09 | 0.23 | 4.58 | 0.53 | 0.12 | 3.7 | 1.08 |

^*^The concentration of protein was 1.6 μM in all cases. The molar ratio of KirBac1.1/total lipid is 1:100.

^#^Calculated using Eq. 8. See Materials and Methods for other details.

**Table S2 Efficiency of spin-labels in quenching the NBD fluorescence of NBD-labeled slide helix mutants of KirBac1.1 in different membranes^#^**

|  | **F/F_o_^*^** | | | |  |
| --- | --- | --- | --- | --- | --- |
| **Residues** | **Tempo-PC** | **5-Doxyl PC** | | **12-Doxyl PC** | **Z_cf_ (Å)** |
| *POPC membranes* |  |  |  |  |  |
| V47 | 0.794 | 0.817 | | 0.956 | 16.3 |
| R49 | 0.766 | 0.826 | | 0.979 | 17.0 |
| D50 | 0.579 | 0.755 | | 0.809 | 19.7 |
| L51 | 0.736 | 0.885 | | 0.926 | 18.6 |
| Y53 | 0.869 | 0.892 | | 0.938 | 16.3 |
| W54 | 0.678 | 0.911 | | 0.981 | 20.5 |
| A55 | 0.663 | 0.846 | | 0.926 | 19.4 |
| K57 | 0.895 | 0.969 | | 0.982 | 17.0 |
| *POPC/POPG membranes* |  |  |  |  |  |
| V47 | 0.976 | 0.741 | 0.982 | | 11.8 |
| R49 | 0.872 | 0.814 | 0.943 | | 14.9 |
| D50 | 0.738 | 0.704 | 0.906 | | 15.2 |
| L51 | 0.753 | 0.801 | 0.948 | | 16.8 |
| Y53 | 0.813 | 0.824 | 0.979 | | 16.1 |
| W54 | 0.806 | 0.744 | 0.824 | | 14.7 |
| A55 | 0.988 | 0.884 | 0.992 | | 14.2 |
| K57 | 0.825 | 0.976 | 0.983 | | 18.3 |

^*^F/F_o_ is the ratio of fluorescence intensity of NBD-labeled mutants in proteoliposome samples in the presence and absence of indicated spin-label. Z_cf_ (Å) is the average distance of NBD group of NBD-labeled mutants from the center of the bilayer, and the distance values are calculated using the F/F_o_ of best quenching pair, which is Tempo-PC and 5-SLPC in our case.

^#^The concentration of protein was 1.6 μM in all cases. The molar ratio of KirBac1.1/ total lipid is 1:100.

The excitation wavelength was 465 nm, and the emission was monitored at respective emission maximum. See Materials and Methods for other details.


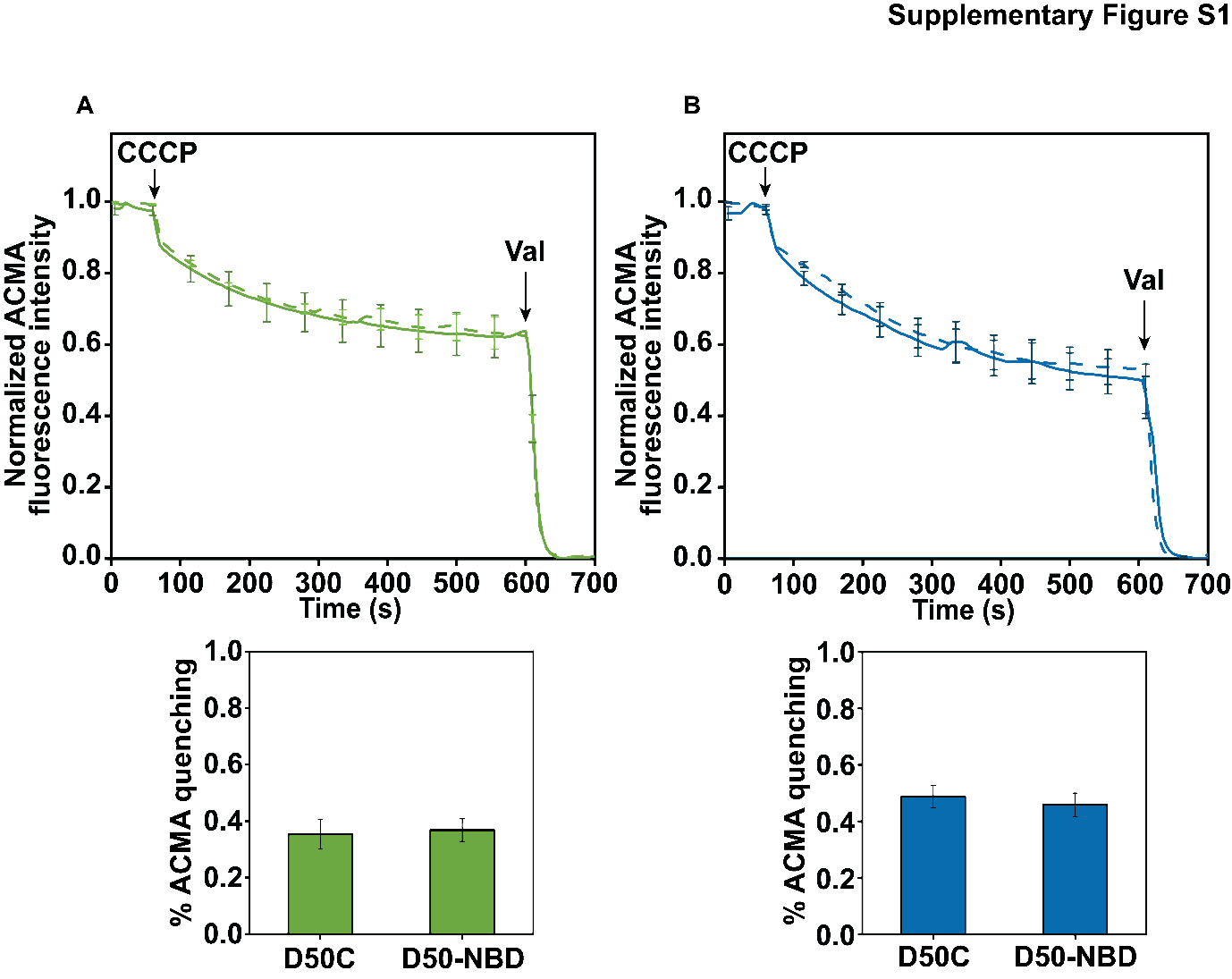


**Figure S1**. NBD-labeling does not affect the PG-dependent gating of KirBac1.1. K^+^ transport assay of D50C (solid trace) and D50-NBD (dotted trace) of KirBac1.1 reconstituted in PC/PG liposomes containing (*A*) 25 mol% and (*B*) 40 mol% POPG lipid. The %ACMA quenching is shown for both the NBD-labeled and unlabeled mutants. Shown are the changes in the normalized fluorescence intensity of pH-sensitive dye ACMA monitored in real time. The concentration of KirBac1.1 was 3.8 μM in all cases. The excitation wavelength used was 410 nm and the emission was monitored at 480 nm. Arrows show the time at which CCCP and valinomycin were added. All data are represented as mean ± SE of three independent measurements. See Materials and Methods for other details.


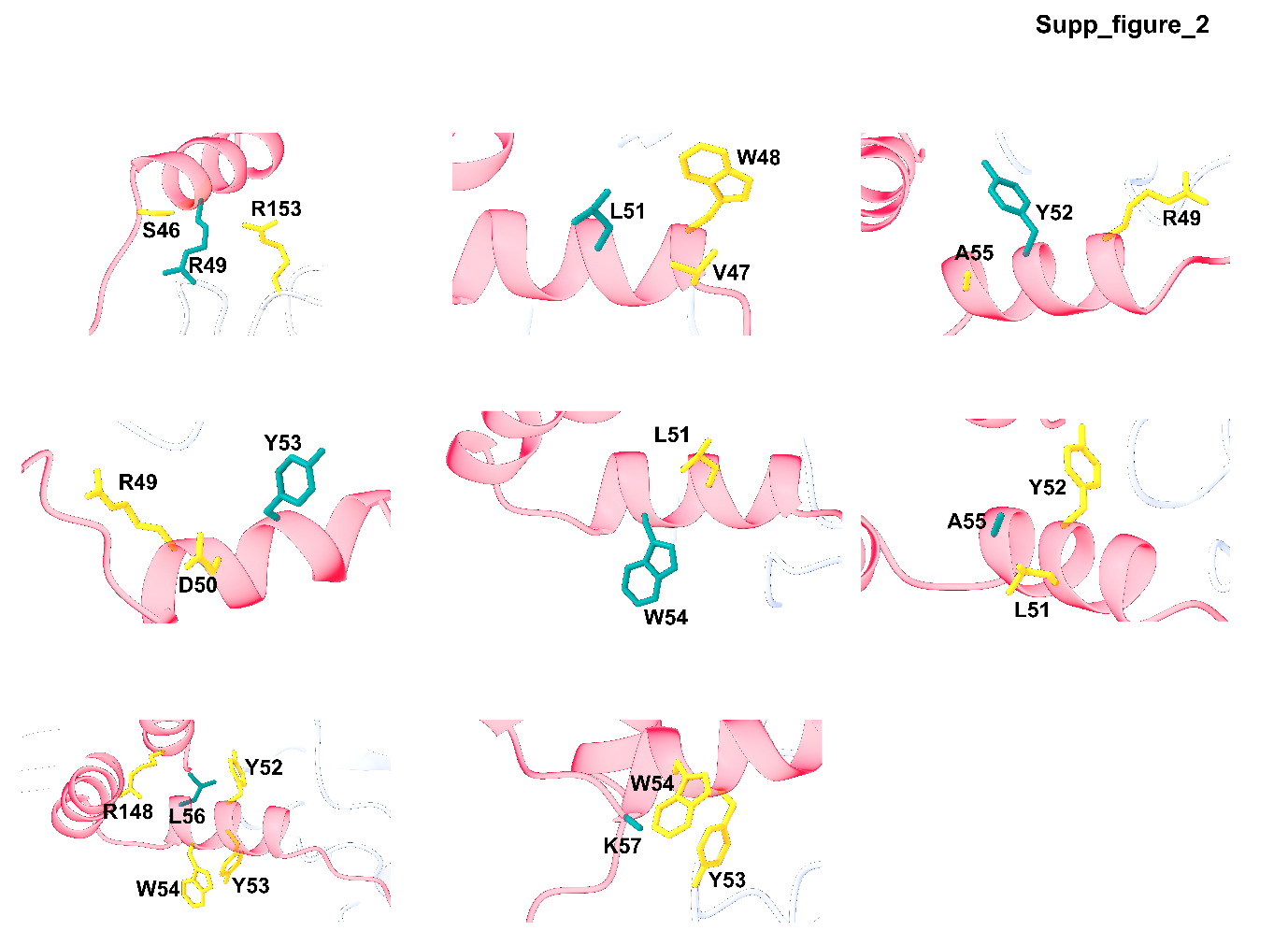


**Figure S2**. Amino acids surrounding slide helix residues. Neighboring residues within 4Ǻ (represented as yellow sticks) of the slide helix residues (represented as blue sticks) are shown based on the closed state structure of KirBac1.1 (*PDB: 2WLL*). The names of the residues are shown as one-letter amino acid code. The close proximity of the presence of charged and aromatic residues, which might act as efficient quenchers of NBD fluorescence and facilitate the decay process, might be responsible for the discrepancy observed between the extent of emission maximum and the mean fluorescence lifetimes for few residues in the slide helix (see main text for details).


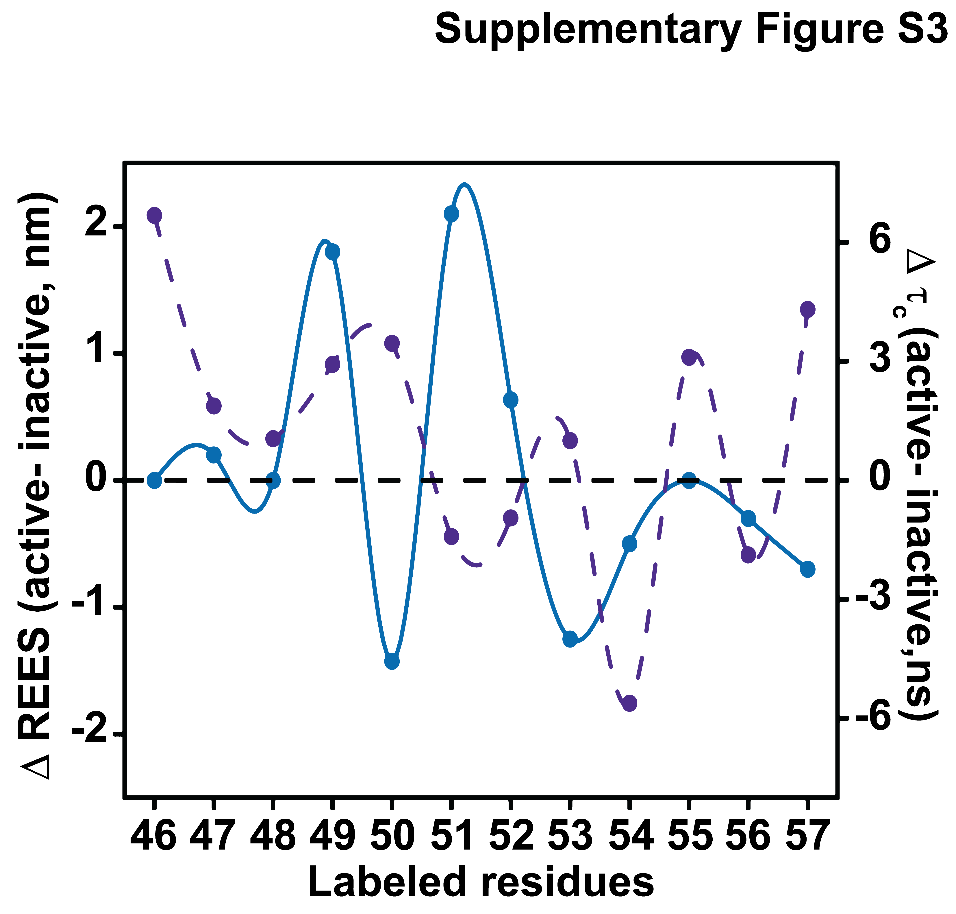


**Figure S3**. Comparison of changes in hydration dynamics and rotational dynamics upon PG-induced gating. The differences in hydration dynamics (ΔREES, shown as solid line) is compared to differences in rotational dynamics (Δτ_c,_ shown as dotted line) for the slide helix residues between the active and inactive conformations of the channel to show the residues that undergo restricted mobility due to PG-driven conformational changes upon gating. The joining lines are provided merely as viewing guides.
